# Computational and AI-Enabled Imaging Biomarkers for Predicting and Assessing Immunotherapy Response in Oral Squamous Cell Carcinoma: A Systematic Review with Functional Meta-Synthesis

**DOI:** 10.3390/medsci14050554

**Published:** 2026-09-08

**Authors:** Carlos M. Ardila, Anny M. Vivares-Builes, Eliana Pineda-Vélez

**Affiliations:** 1 Department of Periodontics, Saveetha Institute of Medical and Technical Sciences, Saveetha Dental College and Hospitals, Saveetha University, Chennai 600077, India; 2 Biomedical Stomatology Research Group, Basic Sciences Department, Faculty of Dentistry, Universidad de Antioquia U de A, Medellín 050010, Colombia; anny.vivares@uam.edu.co (A.M.V.-B.); eliana.pineda@uam.edu.co (E.P.-V.); 3 Faculty of Dentistry, Institución Universitaria Visión de las Américas, Medellín 050040, Colombia

**Keywords:** artificial intelligence, immunotherapy, medical imaging, oral squamous cell carcinoma, radiomics, treatment response

## Abstract

**Background/Objectives:** Computational and artificial intelligence (AI)-enabled imaging biomarkers are increasingly being investigated for immunotherapy-response assessment in oral squamous cell carcinoma (OSCC), but the evidence is heterogeneous. This systematic review identified, critically appraised, and synthesized imaging biomarkers used to predict or assess observed immunotherapy response in OSCC. **Methods:** PubMed/MEDLINE, Embase, and Scopus were searched from inception to 31 July 2026, without language restrictions. The protocol was registered in PROSPERO. Eligible studies evaluated quantitative, radiomic, machine-learning, or related medical-imaging biomarkers against pathological or radiological response. Risk of bias was assessed using design-specific tools, supplemented by a radiomics-specific methodological appraisal. A structured Synthesis Without Meta-analysis was prespecified. **Results:** Seven reports were included, covering pretreatment prediction, early/on-treatment dynamic assessment, and post-neoadjuvant/preoperative response assessment. Pretreatment-computed tomography and magnetic resonance imaging radiomics showed discriminatory performance, whereas baseline positron emission tomography uptake alone was not consistently associated with pathological response. Longitudinal fibroblast activation protein inhibitor positron emission tomography changes were associated with a major pathological response, and magnetic resonance imaging habitat and longitudinal habitat models showed higher within-study discrimination when combined with clinical information. No computational model had independent external validation specifically in an OSCC population, and no three independent studies were sufficiently comparable for meta-analysis. **Conclusions:** Computational imaging is promising across several stages of immunotherapy-response assessment. However, based on current evidence, none of the reviewed imaging biomarkers or models can yet be considered ready for routine clinical use. Prospective multicenter OSCC-specific validation and standardized imaging and analytical pipelines are needed to establish generalizability and clinical utility.

## 1. Introduction

Oral cavity cancer remains a substantial global health problem and represents a major component of cancers arising in the lip, oral cavity, and pharyngeal region. In 2022, approximately 758,000 new cancers of the lip, oral cavity, and pharynx were diagnosed worldwide, with oral cavity tumors accounting for about 42% of this burden [1]. Most malignant tumors in this anatomical region are squamous cell carcinomas, and oral squamous cell carcinoma (OSCC) therefore contributes substantially to oral-cancer morbidity. For resectable diseases, surgery remains central to treatment, often combined with neck dissection and, according to pathological risk features, adjuvant radiotherapy or chemoradiotherapy, with potentially important consequences for speech, swallowing, mastication, and appearance [2]. Programmed cell death protein 1 (PD-1)-directed immunotherapy has transformed systemic treatment of recurrent or metastatic head and neck squamous cell carcinoma (HNSCC), and the phase III KEYNOTE-689 trial recently extended this therapeutic strategy into the perioperative setting by demonstrating improved event-free survival with pembrolizumab added to surgery and risk-adapted adjuvant therapy in resectable locally advanced HNSCC [3]. As immunotherapy moves into potentially curable disease, accurate assessment of treatment response becomes increasingly relevant for patients with oral cavity tumors.

Response to immune checkpoint inhibition, however, remains heterogeneous. Systematic reviews and meta-analyses of neoadjuvant immunotherapy and chemoimmunotherapy in resectable HNSCC have reported meaningful rates of major and complete pathological response, while also showing that many patients do not achieve deep tumor regression [4,5]. Programmed death ligand 1 (PD-L1) combined positive score (CPS) is the most established tissue biomarker used to enrich for benefit from PD-1 blockade, but its predictive ability is limited by spatial and temporal heterogeneity, tissue-sampling and assay-related factors, and incomplete representation of tumor–immune–stromal interactions [6,7]. These limitations provide a rationale for complementary imaging biomarkers that can non-invasively characterize tumor phenotypes and be repeatedly assessed across treatment time points.

The imaging evidence in OSCC has consequently evolved from conventional quantitative measurements toward increasingly complex computational representations. Serial positron emission tomography/computed tomography (PET/CT) studies have evaluated metabolic, PD-L1-targeted, and fibroblast activation protein inhibitor signals before and during immunotherapy, with findings suggesting that longitudinal changes may convey different information from the baseline uptake alone [8,9,10]. Computational approaches have extended this framework through computed tomography (CT) and magnetic resonance imaging (MRI) radiomics, intratumoral and peritumoral modeling, habitat imaging, and longitudinal delta-radiomics [11,12,13,14]. Importantly, these approaches do not address a single clinical task. Pretreatment models aim to predict susceptibility to response before therapy begins, early dynamic biomarkers assess treatment-related change after therapy has started, and post-neoadjuvant/preoperative models attempt to characterize residual response before definitive pathological assessment. These distinctions are clinically important because performance estimates obtained at different treatment stages should not be interpreted as measures of the same prediction problem.

Despite this emerging literature, no previous review has specifically synthesized these imaging-response tasks within an OSCC-focused immunotherapy framework. Existing systematic reviews of neoadjuvant immunotherapy in HNSCC have primarily addressed efficacy, safety, pathological response rates, and candidate tissue or molecular biomarkers [4,5], whereas broader immunotherapy-biomarker reviews emphasize PD-L1, tumor mutational burden, immune-cell populations, transcriptomic signatures, and circulating markers, with imaging considered only as part of a wider biomarker landscape [6,7]. Conversely, a recent systematic review and meta-analysis of MRI radiomics for treatment-response assessment in HNSCC focused on neoadjuvant chemotherapy rather than immunotherapy and included anatomically heterogeneous head and neck populations [15]. Recent reviews of artificial intelligence in oral cancer have likewise concentrated mainly on detection, diagnosis, and risk or prognostic classification rather than response to immune-based treatment [16,17]. The unresolved question is therefore not simply whether imaging can discriminate responders from non-responders, but which imaging approaches have been evaluated for each clinically distinct stage of immunotherapy-response assessment in OSCC, how robustly their performance has been validated, and how close the available evidence is to clinical translation.

This systematic review was therefore designed to identify, critically appraise, and synthesize quantitative, computational, and AI-enabled medical-imaging biomarkers used to predict or assess observed responses to immunotherapy-based treatment in OSCC. We evaluated the evidence according to imaging modality, analytical approach, acquisition timing, treatment context, response definition, and validation level, while also examining calibration, clinical utility, reproducibility, interpretability, and reported biological correlates. Mixed HNSCC studies were considered only when oral-cavity-specific response or model-performance data were separately extractable, thereby preserving directness to the population of interest.

## 2. Materials and Methods

### 2.1. Review Design and Protocol

We conducted and reported this systematic review in accordance with the Preferred Reporting Items for Systematic Reviews and Meta-Analyses (PRISMA) 2020 statement [18]. We developed the protocol a priori following PRISMA-P principles [19] and registered it in the International Prospective Register of Systematic Reviews (PROSPERO; CRD420261482259). We designed the review to evaluate medical-imaging biomarkers that provide quantitative or computational information about observed responses to immunotherapy-based treatment in OSCC while preserving the distinction between conventional quantitative imaging, radiomics, and machine-learning or deep-learning approaches. Because we expected the available evidence to differ substantially in imaging modality, analytical strategy, timing of image acquisition, treatment setting, and response definition, we prespecified a structured synthesis as the primary analytical approach and allowed exploratory quantitative pooling only when clinically and methodologically defensible.

### 2.2. Eligibility Criteria

The review question was: in adults with histologically confirmed OSCC or OCSCC receiving an immunotherapy-containing treatment regimen, what quantitative, computational, radiomic, machine-learning, or deep-learning medical-imaging biomarkers have been evaluated for prediction or assessment of observed treatment response, and what is their predictive performance and methodological robustness? Eligibility was defined using a Participants, Intervention/Exposure, Comparators, Outcomes, and Study design (PICOS) framework, with imaging timing incorporated as an additional prespecified dimension.

Participants (P): Adults with histologically confirmed primary OSCC or OCSCC at any clinically relevant disease stage, including resectable locally advanced, recurrent, or metastatic disease, were eligible. Mixed HNSCC cohorts were considered only when oral-cavity-specific response estimates or model-performance data were separately extractable; this restriction was used to maintain directness to the population specified in the review question.

Intervention or exposure (I): Eligible exposures were medical imaging-derived quantitative, computational, or AI-enabled biomarkers evaluated in patients receiving an immunotherapy-containing regimen. These included CT-based quantitative imaging and radiomics; MRI, diffusion-weighted imaging, and derived quantitative parameters; PET/CT and molecular PET imaging; intratumoral or peritumoral radiomics; habitat and delta-radiomics approaches; machine-learning and deep-learning models; and multimodal imaging–clinical models. Imaging could be obtained before treatment, during or shortly after immunotherapy, or after completion of neoadjuvant treatment but before definitive pathological response ascertainment. These time points were retained separately because a pretreatment predictor, an early dynamic biomarker, and a preoperative response-assessment biomarker answer different clinical questions.

Comparators (C): A separate untreated or active-treatment control group was not required. Eligible comparisons included responders versus non-responders, pCR versus non-pCR, MPR versus non-MPR, radiological responders versus non-responders, alternative imaging models, clinical models, and pathological biomarkers such as PD-L1 CPS when these were used as comparators for imaging-based prediction.

Outcomes (O): The primary outcome was an observed response to an immunotherapy-containing regimen. Eligible endpoints included pCR, MPR, percentage pathological tumor regression or comparable pathological response categories, and radiological response according to the Response Evaluation Criteria in Solid Tumors (RECIST), immune RECIST (iRECIST), or another explicitly defined framework. Extractable predictive-performance measures included the area under the receiver operating characteristic curve (AUC)/c-statistic, sensitivity, specificity, accuracy, predictive values, likelihood ratios, and correlation or regression estimates relating an imaging biomarker to response, together with confidence intervals when reported. Pathological and radiological response outcomes were not considered interchangeable. Secondary outcomes included calibration, decision-curve analysis (DCA), net clinical benefit, validation level, reproducibility, model interpretability, incremental performance over clinical or pathological biomarkers, relevant biological correlates, and progression-free survival (PFS) or overall survival (OS) when directly linked to the evaluated response-prediction model.

Study design (S): Original human studies reporting sufficient information to evaluate an eligible imaging biomarker or prediction model were included. Eligible designs comprised prospective and retrospective cohorts, secondary imaging analyses of clinical trials, prospective imaging-biomarker studies, and model-development and/or validation studies. Only full-text original reports contributed to the primary synthesis. Conference abstracts without a corresponding full-text report were retained only as supplementary emerging evidence and were not used for the primary synthesis or any quantitative pooling because their abbreviated reporting generally precludes a complete methodological appraisal.

### 2.3. Exclusion Criteria

Studies were excluded if they evaluated non-oral primary tumors or mixed HNSCC populations without separately extractable oral-cavity-specific imaging-response or model-performance results; used animal or in vitro data; assessed imaging only for diagnosis, anatomical staging, surveillance, segmentation, or conventional visual response evaluation without a quantitative/computational biomarker; predicted PD-L1 expression, immune-cell infiltration, molecular signatures, recurrence, prognosis, lymph-node metastasis, grading, or other biological endpoints without an observed immunotherapy-response outcome; used exclusively histopathology, pathomics, genomic, transcriptomic, or other non-medical-imaging information; or evaluated treatment without an immunotherapy component. Reviews, meta-analyses, editorials, commentaries, case reports, protocols without results, and non-full-text conference reports were also excluded from the primary evidence set.

### 2.4. Information Sources and Search Strategy

We searched PubMed/MEDLINE, Embase, and Scopus from database inception to 31 July 2026, without publication-date or language restrictions. We followed the principles of the PRISMA-S extension [20] when reporting the search. We used Google Scholar as a supplementary source and screened the first 200 records ranked by relevance. We also performed backward reference-list checking and forward citation searching for included studies and relevant reviews and searched ClinicalTrials.gov and other study registers to identify linked publications, companion reports, and potentially overlapping trial cohorts. We did not include registry-only unpublished results as primary evidence.

The database strategies combined three core concepts: (1) OSCC/OCSCC, (2) quantitative or computational medical imaging, and (3) immunotherapy or immune checkpoint blockade. For example, the PubMed/MEDLINE search included combinations of terms such as *“Mouth Neoplasms” OR “oral squamous cell carcinoma” OR OSCC*, “Radiomics” OR radiomic* OR “artificial intelligence” OR “machine learning” OR PET OR “magnetic resonance imaging”, and “Immunotherapy” OR immunotherap* OR “immune checkpoint inhibitor*” OR PD-1 OR PD-L1. To reduce the risk of missing mixed cohorts indexed only under HNSCC, each database strategy also incorporated a complementary HNSCC sensitivity component that combined HNSCC terminology with computational-imaging and treatment-response terms. Records identified through this sensitivity component remained subject to the prespecified requirement for separately extractable oral-cavity-specific response or model-performance data. The complete database-specific search strategies, including controlled vocabulary, field tags, Boolean operators, and search yields, are provided in Supplementary Table S1.

### 2.5. Selection Process

Two reviewers independently screened titles and abstracts against the prespecified eligibility criteria. Reports considered potentially relevant by either reviewer underwent independent full-text assessment. We resolved disagreements by discussion, with adjudication by a third reviewer when consensus could not be reached. Reasons for exclusion at the full-text stage were recorded and are presented in Supplementary Table S2. Inter-reviewer agreement for categorical selection decisions was high (Cohen’s κ = 0.90). The study-selection process was documented using the PRISMA 2020 flow diagram [18].

### 2.6. Data Collection Process

Two reviewers independently extracted data using a standardized, prepiloted form (Supplementary Table S3). We developed the extraction framework using the CHARMS checklist for systematic reviews of prediction-model studies [21] and expanded it to capture imaging-specific characteristics and single quantitative biomarker studies. We resolved discrepancies by consensus or, when necessary, through adjudication by a third reviewer. We assessed inter-reviewer agreement according to the scale of the extracted variable: agreement for categorical decisions was κ = 0.90 and agreement for continuous numerical data was ICC = 0.91.

No author contact was required because the information necessary for eligibility assessment and data extraction was available from the published reports and Supplementary Materials.

### 2.7. Data Items

Extracted study-level information included authors, publication year, country, recruiting institutions, recruitment period, study design, prospective or retrospective status, trial registration identifiers, sample size, disease setting, anatomical oral subsite, tumor stage, eligibility criteria, and treatment regimen. Imaging variables included modality, scanner characteristics when available, acquisition sequence or tracer, timing in relation to treatment and surgery, segmentation strategy, region or volume of interest, image preprocessing, quantitative imaging parameters, radiomic feature families, feature-selection procedures, habitat or delta-feature derivation, and software used for image analysis.

For computational models, data were collected on candidate predictors, final feature set, algorithm or model class, hyperparameter tuning, handling of class imbalance and missing data, training/test splitting, cross-validation or bootstrap procedures, internal and external validation, model calibration, decision-curve analysis, and interpretability methods. Training, internal-validation, held-out test, and external-validation performance estimates were extracted separately rather than being treated as equivalent. Outcome data included the definition and timing of treatment response, the reference standard, numbers of responders and non-responders, and all reported discrimination or classification measures with uncertainty estimates. Funding, conflicts of interest, protocol availability, biological correlates, and information relevant to cross-publication participant overlap were also recorded.

### 2.8. Management of Multiple Reports and Participant Overlap

We examined potential participant overlap before combining any participant totals or quantitative estimates. We compared recruitment institutions, recruitment windows, trial identifiers, inclusion criteria, treatment regimens, imaging protocols, authorship, and reported cohort characteristics across eligible reports. We linked multiple reports when they clearly originated from the same trial or source population. When overlap was possible but could not be quantified at the participant level, we retained reports that contributed distinct eligible imaging analyses but treated them as potentially non-independent evidence. We did not allow such reports to contribute simultaneously to the same pooled denominator or exploratory meta-analysis. We constructed a cross-publication overlap matrix to document these judgments. When published aggregate information was insufficient to establish independence, we recorded the specific information that would have been required for definitive linkage across reports, including center-specific enrollment information, precise recruitment timing, source-cohort or trial identifiers, and de-identified participant-level linkage information where available.

### 2.9. Study Risk of Bias and Methodological Quality Assessment

A design-matched appraisal strategy was used because the evidence base contained both multivariable prediction models and studies of individual quantitative imaging biomarkers. Multivariable regression, radiomics, machine-learning, and deep-learning prediction-model studies were assessed using PROBAST+AI [22]. For model-development studies, the PROBAST+AI model-development component was applied; for studies evaluating a previously developed model or algorithm, the model-evaluation component was used. When a report contained both development and evaluation, the relevant components were considered separately so that internal model construction was not conflated with independent performance assessment.

Studies evaluating individual quantitative imaging biomarkers without development of a multivariable prediction model were assessed using the Quality In Prognosis Studies (QUIPS) framework [23], treating the imaging measure as the candidate predictor and observed treatment response as the subsequent outcome. In addition, radiomics and AI-based studies were evaluated with the METhodological RadiomICs Score (METRICS) as a complementary assessment of radiomics-specific methodological quality [24]. METRICS was not used as a substitute for risk-of-bias assessment and no study was excluded on the basis of its score.

All assessments were performed independently by two reviewers, followed by consensus. Inter-reviewer agreement for categorical risk-of-bias judgments was κ = 0.90, whereas agreement for continuous METRICS scoring was ICC = 0.91. Risk-of-bias judgments and methodological-quality results were summarized at study and domain/item level and were incorporated into the interpretation of the evidence rather than used as a simple numerical weighting scheme.

### 2.10. Reporting Bias Assessment

Selective outcome and analysis reporting were assessed qualitatively by comparing full publications with trial registrations, protocols, conference reports, and companion publications when these were available. Particular attention was given to discrepancies in prespecified response outcomes, imaging time points, model-selection procedures, and reported performance metrics. Formal assessment of small-study effects by funnel plot or regression-based tests was prespecified only if at least 10 sufficiently comparable and independent studies contributed to the same quantitative synthesis; otherwise, publication and selective-reporting bias were considered descriptively.

### 2.11. Confidence in the Evidence

Consistent with the registered protocol, a formal GRADE rating was not imposed across the entire evidence base. The review combined prediction-model studies and single quantitative imaging-biomarker studies, and the objective was not to estimate a single intervention effect. Confidence was therefore characterized transparently within each synthesis domain by considering risk of bias, directness to OSCC/OCSCC, consistency of findings, precision, sample size, validation level, generalizability across centers and imaging platforms, potential reporting bias, and independence of contributing cohorts. External validation was distinguished explicitly from held-out testing and internal resampling, and evidence from potentially overlapping populations was interpreted as non-independent.

### 2.12. Data Synthesis and Statistical Analysis

The primary synthesis used a structured functional meta-synthesis informed by the applicable elements of the Synthesis Without Meta-analysis (SWiM) reporting guideline [25]. SWiM was used to improve transparency in grouping, presentation, and interpretation of heterogeneous quantitative evidence rather than as a statistical model. The functional synthesis proceeded in four steps. First, study-, imaging-, treatment-, and outcome-level data were standardized so that differences in terminology did not obscure clinically similar constructs. Second, studies were grouped into clinically and methodologically coherent evidence domains after extraction using prespecified factors such as imaging modality and analytical approach, imaging timing, treatment context, response definition, and validation level; no common effect measure was imposed when these factors were not sufficiently aligned. Third, within each group, the direction and magnitude of imaging-response associations, discrimination and classification performance, calibration, validation, clinical utility, and methodological limitations were compared. Finally, findings were integrated across groups to identify convergent and discordant patterns, evaluate the extent to which performance was reproduced beyond model-development data, and determine where evidence remained insufficient for clinical translation. Statistical significance alone was not used for vote counting.

Exploratory quantitative synthesis was prespecified but was not automatic. A meta-analysis would be considered only when at least three independent studies were sufficiently comparable in population, treatment setting, imaging modality or analytical class, imaging time point, response definition, and performance metric, in line with methodological guidance for systematic reviews of prediction-model performance [26]. One estimate per independent cohort would contribute to a given analysis. When a study reported several models, the model designated as primary by the authors was preferred; when no primary model was specified, the estimate with the highest level of validation (external validation, held-out test, internal validation/resampling, then development performance) was selected rather than choosing the numerically largest AUC. Potentially overlapping reports were not pooled together.

For sufficiently comparable AUC/c-statistic estimates, validation or test-set estimates would be transformed to an appropriate unbounded scale, preferentially the logit scale, pooled using a random-effects model, and back-transformed for presentation; this approach was chosen because raw c-statistics are bounded and may violate between-study normality assumptions [27]. Random-effects variance would be estimated by restricted maximum likelihood, with 95% confidence intervals and, when statistically meaningful, prediction intervals. Heterogeneity would be described using τ^2^ and I^2^ but interpreted cautiously when few studies were available. If sufficiently comparable sensitivity and specificity estimates could be reconstructed at compatible thresholds, a bivariate random-effects model would be considered; otherwise, these measures would be summarized without pooling. Training-set performance was not pooled when a validation or test estimate was available from the same study, and pathological response and radiological-response outcomes were analyzed separately.

Sensitivity analyses, when feasible, were planned to exclude studies at high risk of bias, studies lacking independent validation, and one member of any potentially overlapping report pair. Meta-regression and formal subgroup testing were reserved for situations with an adequate number of independent studies and were not to be undertaken simply to explore sparse data. Quantitative analyses were planned in R version 4.6.1 (R Foundation for Statistical Computing, Vienna, Austria), principally using the metafor package for random-effects pooling and the mada package if bivariate sensitivity/specificity synthesis became appropriate. If the prespecified comparability criteria were not met, no pooled estimate would be produced and the SWiM-informed functional meta-synthesis would constitute the final synthesis.

## 3. Results

### 3.1. Study Selection

The database searches identified 653 records, comprising 154 from PubMed/MEDLINE, 286 from Scopus, and 213 from Embase. After removal of 247 duplicate records, 406 unique records remained for title and abstract screening. Of these, 393 were excluded, and 13 reports were retrieved and assessed in full text. Six reports were excluded at the full-text stage. Five evaluated imaging biomarkers or predictive models in mixed HNSCC populations that included oral-cavity tumors but reported treatment-response associations or model-performance estimates only for the overall HNSCC cohort; because oral-cavity-specific results could not be separately extracted, these reports did not meet the prespecified population criterion. The remaining report enrolled patients with locally advanced HNSCC but contained no eligible oral-cavity primary tumors in the analyzed cohort. Full references and study-specific reasons for exclusion are provided in Supplementary Table S2.

**Figure 1:**
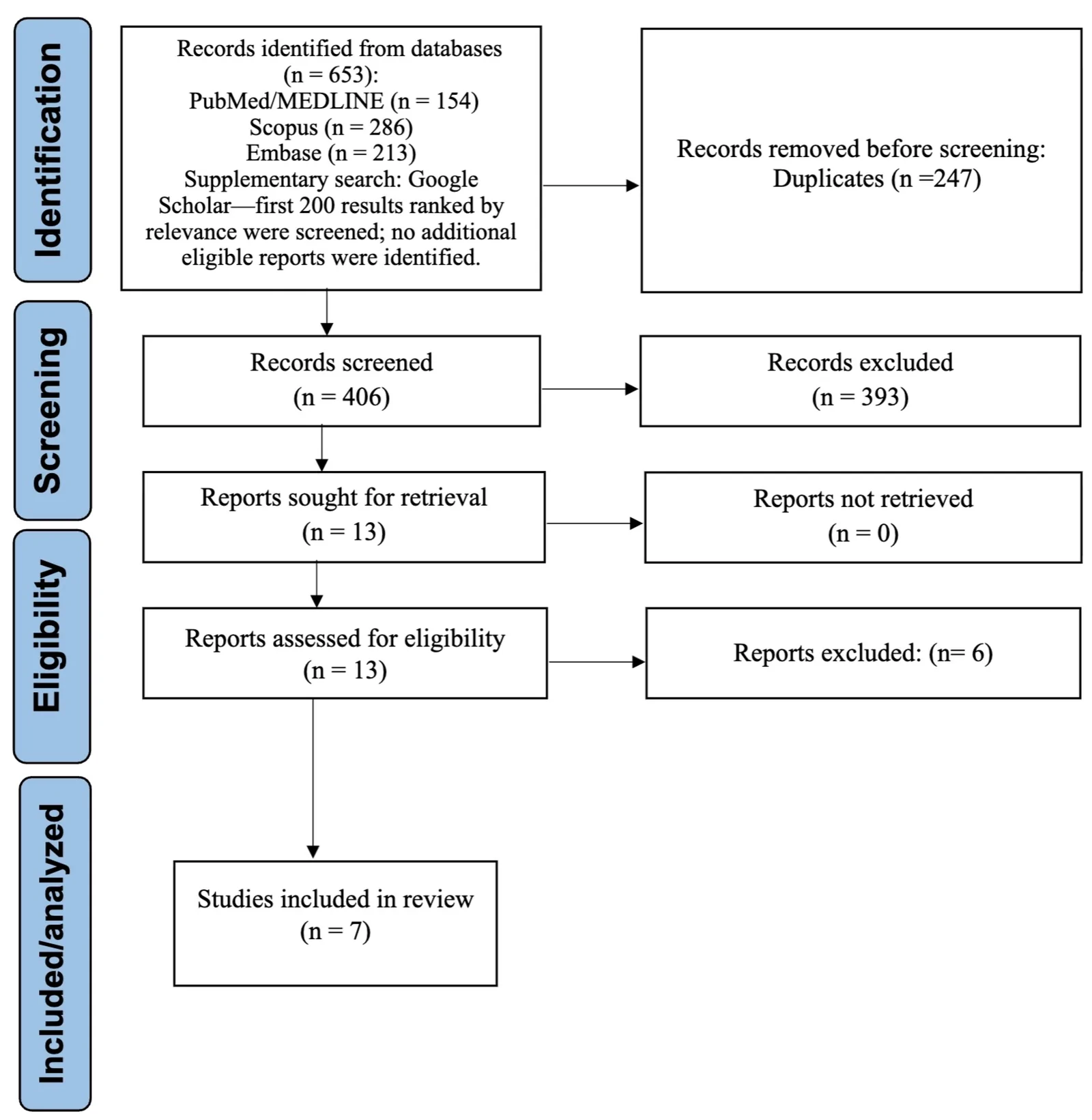
PRISMA 2020 flow diagram of the study-selection process.

Seven reports fulfilled the eligibility criteria and were included in the systematic review. Supplementary screening of the first 200 Google Scholar records ranked by relevance, backward and forward citation searching, and searches of trial and study registers identified no additional eligible reports. The complete study-selection process is summarized in the PRISMA 2020 flow diagram (Figure 1).

### 3.2. Characteristics of Included Reports and Study Populations

Seven eligible reports published between 2022 and 2026 were included [8,9,10,11,12,13,14]. The evidence was geographically concentrated, with five reports conducted in China, one in the United States, and one in the Netherlands. The study designs comprised four retrospective cohort or model-development studies, two prospective biomarker studies, and one retrospective imaging analysis based on prospectively collected data from a randomized phase II trial. Six reports enrolled exclusively patients with OSCC or OCSCC, whereas Lin et al. [12] evaluated a broader HNSCC population but reported treatment-response model performance separately for the oral SCC subgroup; only the oral-specific results from that report were considered in the present review.

The clinical context was predominantly perioperative. Six reports evaluated patients receiving neoadjuvant immunotherapy or NACI followed by definitive surgery, whereas Ma et al. [11] included both locally advanced and recurrent/metastatic OSCC treated with PD-1 inhibitors without surgery during immunotherapy. Accordingly, pathological tumor response after surgery constituted the reference outcome in six reports, while Ma et al. classified response from serial imaging using immune-response categories. The size of the evaluable cohorts varied substantially, from 16 to 212 patients. Study-level population and treatment characteristics are summarized in Table 1.

Potential partial participant overlap was identified between Lin et al. [12] and Ding et al. [14], because both reports included patients treated at Sun Yat-sen Memorial Hospital during substantially overlapping recruitment periods. Lin et al. [12] reported 90 oral SCC cases relevant to this review, of whom 61 were included in the Sun Yat-sen Memorial Hospital training/internal-validation cohorts and could therefore potentially overlap with the 195-patient single-center OCSCC cohort reported by Ding et al. [14]. The remaining 29 oral SCC cases in Lin et al. [12] were recruited from external centers and could not overlap with the Ding et al. cohort. The published reports did not provide participant-level linkage information or an explicit cross-publication statement confirming whether individual patients were shared between these analyses. Definitive assessment of independence would require center-specific enrollment information that permits participant linkage across publications, such as de-identified participant identifiers or equivalent linkage variables, together with sufficiently precise recruitment dates and source-trial or cohort identifiers. In the absence of such information, the extent of actual overlap could not be established. Both reports were therefore retained because they evaluated distinct imaging strategies, but they were treated as potentially non-independent evidence. Consequently, participant numbers were not summed across all seven reports to generate an overall pooled sample size. The cross-publication assessment is detailed in Supplementary Table S4.

### 3.3. Imaging and Computational Approaches

The included reports encompassed distinct levels of imaging complexity, ranging from serial semiquantitative PET measurements to high-dimensional radiomics and spatially and temporally resolved habitat models. Three studies evaluated longitudinal PET-based biomarkers without developing multivariable prediction models [8,9,10], whereas four used radiomic or machine-learning approaches based on CT or MRI [11,12,13,14]. Within the latter group, the imaging task also differed according to acquisition timing: Ma et al. [11] and Lin et al. [12] developed pretreatment predictors, Yuan et al. [13] evaluated habitat features from MRI obtained after NACI and before pathological response ascertainment, and Ding et al. [14] explicitly modeled longitudinal changes between pre- and post-NACI MRI.

The PET studies relied on serial changes in tracer uptake. Shah et al. [8] quantified FDG uptake in the primary tumor and cervical lymph nodes before and shortly after preoperative immunotherapy, including absolute and percentage changes in SUVmax. Wondergem et al. [9] expanded this approach by combining metabolic FDG imaging with PD-L1-targeted PET using ^18F-BMS-986192, thereby providing both metabolic and target-specific measurements across treatment. Jiang et al. [10] used ^68Ga-FAPI-04 PET/CT at baseline and after two cycles of NACI, with longitudinal changes calculated from SUVmax, SUVmean, and SUVpeak. None of these studies applied radiomic feature extraction or machine-learning model development.

The computational studies used different radiomic representations, including conventional tumor radiomics, intratumoral and peritumoral radiomics, habitat imaging, and longitudinal DeltaHabitat features. Ma et al. [11] extracted handcrafted features from a two-dimensional pretreatment CT tumor section and used LASSO-based feature selection before constructing several machine-learning classifiers. Lin et al. [12] used pretreatment multicenter MRI to derive intratumoral and concentric peritumoral radiomic signatures, subsequently integrating imaging information with clinicopathological variables. Yuan et al. [13] partitioned both intratumoral and peritumoral regions into K-means-defined habitats across multiple MRI sequences, whereas Ding et al. [14] combined habitat analysis with delta-radiomics to quantify changes in subregional imaging phenotype between pre- and post-treatment examinations. The technical characteristics of the quantitative PET biomarker studies are summarized in Table 2, whereas the imaging and computational characteristics of the radiomics and machine-learning studies are presented separately in Table 3.

### 3.4. Treatment-Response Prediction by Imaging Biomarkers and Models

The primary treatment-response outcome varied across the included reports. Six studies evaluated imaging findings against postoperative pathological response [8,9,10,12,13,14], whereas Ma et al. [11] assessed radiological response according to iRECIST. Pathological outcomes included continuous tumor regression, MPR, and pCR; these endpoints were retained according to the definitions used in the original reports.

The PET-based studies evaluated response primarily through longitudinal changes in tracer uptake. Shah et al. [8] reported no significant association between early changes in primary-tumor FDG uptake and subsequent pathological response. Wondergem et al. [9] found no significant baseline separation according to PD-L1-targeted PET uptake, although treatment-related FDG changes were observed among pathological responders. Jiang et al. [10] reported significant discrimination of MPR using longitudinal changes in FAPI uptake, whereas baseline FAPI measurements were not significantly associated with response.

Among pretreatment computational approaches, Ma et al. [11] evaluated several CT-radiomics classifiers for iRECIST-defined response, with the neural-network model yielding the highest reported discrimination. Lin et al. [12] evaluated pretreatment MRI radiomics for pCR after NACI and provided separately extractable performances for the oral SCC subgroup.

The habitat-based MRI studies addressed response after treatment initiation. Yuan et al. [13] evaluated intratumoral and peritumoral habitat features obtained after NACI and before surgery, whereas Ding et al. [14] incorporated longitudinal changes between pre- and post-NACI MRI examinations. Both studies reported performances for imaging-only and integrated imaging–clinical approaches. Response definitions, response distributions, and the corresponding study-level performance estimates are presented in Table 4.

### 3.5. Validation, Calibration, Clinical Utility, and Interpretability

Validation procedures differed across the four computational-model reports [11,12,13,14]. Ma et al. [11] used LOOCV without an independent test cohort, whereas Yuan et al. [13] used five-fold cross-validation. Ding et al. [14] evaluated performance in a same-center held-out test set. Lin et al. [12] included training, internal-validation, and external-validation cohorts for the overall HNSCC model; however, external-validation performance was not reported separately for the oral SCC subgroup. The validation framework of each study is detailed in Table 4.

Calibration was reported in three of the four computational-model studies [12,13,14], while Ma et al. [11] did not report a calibration assessment. None provided external calibration specifically in an independent OSCC/OCSCC population. Formal clinical-utility assessment using DCA was reported by Ma et al. [11], Lin et al. [12], and Ding et al. [14], whereas Yuan et al. [13] did not report a net-benefit analysis. None of the included reports prospectively evaluated the effect of model-guided decision making on patient management or clinical outcomes.

Explicit interpretability analyses were reported in two MRI radiomics studies. Lin et al. [12] examined associations between radiomic features and digitally quantified histopathological characteristics, whereas Yuan et al. [13] used SHAP-based feature attribution and investigated relationships with tissue immune characteristics. Dedicated post hoc explainability methods were not reported by Ma et al. [11] or Ding et al. [14].

Manual tumor delineation was used across the computational imaging studies, generally with radiologist review or confirmation. Quantitative segmentation or feature repeatability was not consistently reported, and the longitudinal DeltaHabitat approach additionally required registration of serial MRI examinations. Study-specific information on validation, calibration, clinical utility, interpretability, and reproducibility is summarized in Table 5.

### 3.6. Biological Correlates and Additional Outcomes

Biological correlates related to treatment response or imaging features were reported in four of the seven included reports [9,10,12,13]. Wondergem et al. [9] evaluated peripheral immune-cell phenotypes in addition to serial PET imaging. Patients achieving MPR had higher baseline activation of circulating CD8+ T cells, characterized by higher expression of PD-1, TIGIT, and IFNγ and lower expression of PD-L1. These immune-cell findings were reported alongside the observed post-nivolumab reduction in FDG uptake in the major responders.

Jiang et al. [10] assessed FAP, CD8, GZMB, and PD-L1 by immunohistochemistry in postoperative tumor specimens from 11 of the 20 patients included in the imaging analysis. FAP expression was positively correlated with preoperative FAPI uptake, including SUVmax (*r* = 0.720, *p* < 0.05), SUVmean (*r* = 0.720, *p* < 0.05), and SUVpeak (*r* = 0.736, *p* < 0.01). PD-L1 expression showed a similar positive association with preoperative PET parameters. CD8 expression was positively correlated with GZMB (*r* = 0.873, *p* < 0.01), whereas neither CD8 nor GZMB was significantly correlated with the PET parameters. Compared with the non-MPR group, patients with MPR showed higher CD8 and GZMB expression and lower FAP and PD-L1 expression.

Lin et al. [12] examined pretreatment biopsy samples from 38 patients and compared intratumoral and peritumoral radiomic features with digitally quantified pathological characteristics. Associations were reported between selected radiomic features and immune-cell density, fibroblast density, and TIL-related ratios. These analyses were performed in a subset of the overall HNSCC cohort and were not reported separately for the oral SCC subgroup.

Yuan et al. [13] investigated the biological associations of the habitat features identified by SHAP. Three intratumoral habitat features and one peritumoral habitat feature differed significantly according to the PD-L1 CPS category (*t* = 2.027–2.275, *p* < 0.05). Two peritumoral habitat features were strongly correlated with stromal CD45+ cell density (*r* = 0.958 and −0.920, respectively; *p* < 0.05). PD-L1 CPS was available for 33 patients in that cohort.

No included report provided PFS or OS analyses that were directly linked to an eligible oral-cavity-specific imaging response-prediction model. Accordingly, these prespecified secondary outcomes did not contribute additional results to the synthesis.

### 3.7. Study Risk of Bias and Methodological Quality

**Figure 2:**
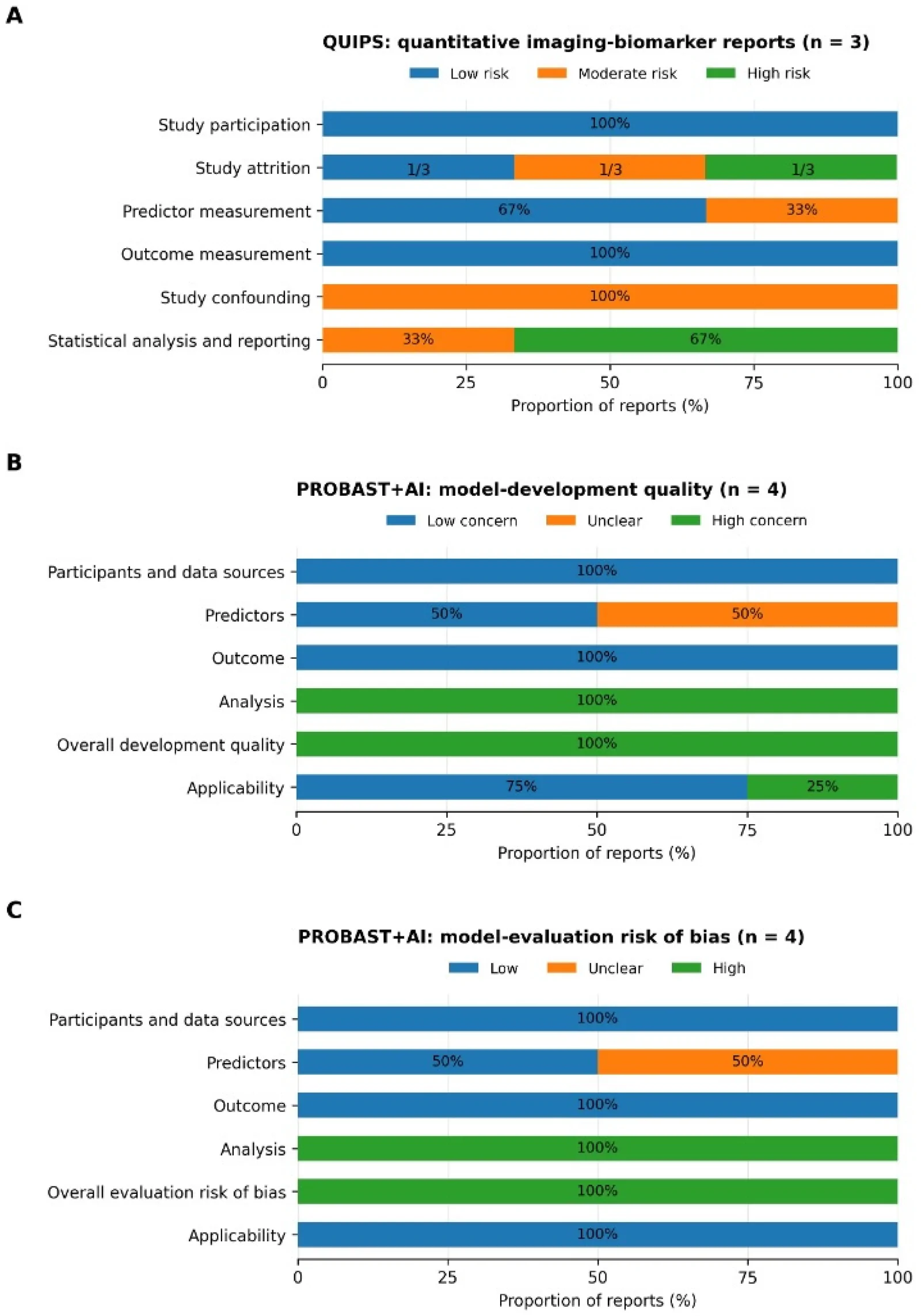
Domain-level risk-of-bias summary of the included quantitative imaging-biomarker and prediction-model reports. Panel (**A**) summarizes the QUIPS assessment of reports evaluating individual quantitative imaging biomarkers. Panel (**B**) summarizes the PROBAST+AI assessment of model-development quality, and Panel (**C**) summarizes the PROBAST+AI assessment of model-evaluation risk of bias. Notably, the analysis domain was rated as high concern for model development and high risk of bias for model evaluation in all four prediction-model reports. Bars represent the proportion of reports assigned to each judgment category within each domain. Detailed report-level judgments and their rationale are provided in Supplementary Table S5. For the QUIPS study-attrition domain, each judgment category comprised one of three reports (1/3); percentages are subject to rounding. (Abbreviations: PROBAST+AI, Prediction model Risk Of Bias ASsessment Tool plus Artificial Intelligence; QUIPS, Quality In Prognosis Studies).

The three reports evaluating individual quantitative PET biomarkers [8,9,10] were assessed with QUIPS, whereas the four radiomics or machine-learning prediction-model reports [11,12,13,14] were assessed with PROBAST+AI. The domain-level distribution of these assessments is summarized in Figure 2, whereas detailed study-level judgments are provided in Supplementary Table S5. In the QUIPS assessment, study participation and outcome measurement were rated at low risk of bias in all three reports. Shah et al. [8] was rated at moderate risk for predictor measurement, study confounding, and statistical analysis and reporting. Wondergem et al. [9] had moderate risk related to attrition and confounding and high risk in statistical analysis and reporting. Jiang et al. [10] was rated at high risk for attrition and statistical analysis and reporting and at moderate risk for confounding. Shah et al. [8] included 27 evaluable participants, with five baseline PET examinations acquired at outside institutions, whereas Wondergem et al. [9] included 16 evaluable patients, only three of whom achieved MPR. Jiang et al. [10] enrolled 31 patients but included 20 in the final imaging-response analysis.

For model development, PROBAST+AI identified low concern in the participants/data-sources and outcome domains for all four prediction-model reports, whereas the analysis domain was rated at high concern in each report. Predictor-domain concern was low for Ma et al. [11] and Ding et al. [14] and unclear for Lin et al. [12] and Yuan et al. [13], primarily because the available reports did not provide sufficient information to determine whether all predictor-assessment procedures were performed without knowledge of the outcome. The overall model-development quality judgment was therefore high concern for all four reports. Applicability concern for model development was low for Ma et al. [11], Yuan et al. [13], and Ding et al. [14] and high for Lin et al. [12], because its model-development population comprised mixed HNSCC rather than an exclusively oral-cavity population. Lin et al. [12] nevertheless reported separately extractable performance for the oral SCC subgroup, as required by the eligibility criteria of this review.

In the model-evaluation component of PROBAST+AI, the analysis domain and overall risk of bias were rated high for all four reports. Ma et al. [11] used LOOCV, but the published workflow did not establish that the complete feature-selection procedure was repeated within each resampling iteration. Lin et al. [12] included separate training, internal-validation, and external-validation cohorts for the overall HNSCC model; however, the eligible oral SCC performance estimate was reported as a subgroup result rather than as a separately reported external oral-cavity validation estimate. Yuan et al. [13] used five-fold cross-validation, although the reported workflow did not establish that clustering, imputation, and feature-selection procedures were fully repeated within each cross-validation fold. Ding et al. [14] used a held-out 7:3 test partition, but the random partitioning was repeated to obtain approximately balanced baseline characteristics. Evaluation applicability to the review question was rated at low concern for all four reports.

Taken together, the recurring analysis-domain concerns across the four prediction-model reports involved limited sample size relative to model complexity, incomplete evidence that feature selection and other data-driven preprocessing steps were fully contained within resampling procedures, and evaluation strategies that did not establish independent OSCC/OCSCC-specific external validity. In some studies, the independence of data partitioning was also uncertain. These limitations increase susceptibility to overfitting and optimistic performance estimates and therefore reduce confidence that the reported discrimination would be reproducible in new patients or across institutions.

METRICS ranged from 38.3% to 88.5% (Supplementary Table S6). Ma et al. [11] scored 38.3% and was categorized as low methodological quality; Lin et al. [12] scored 88.5% and was categorized as excellent; Yuan et al. [13] scored 49.9%; and Ding et al. [14] scored 59.5%, both within the moderate category. At the item level, recurring weaknesses included inadequate or unclear early data partitioning and leakage prevention, incomplete reporting of preprocessing and feature-extraction parameters, limited external testing, and restricted public availability of data, code, and models. Robustness and reproducibility were also incompletely addressed: removal of non-robust features was reported in only two studies, and quantitative segmentation or feature-repeatability assessment was not consistently documented. Calibration and uncertainty estimates were reported in three studies, but no study provided independent OSCC-specific external calibration. The complete item-level METRICS assessment and weighted scores are presented in Supplementary Table S6.

### 3.8. Reporting Bias and Confidence in the Evidence

Prospective registration or protocol documentation relevant to the imaging-response analyses was available to different degrees across the included reports. Shah et al. [8] was a secondary retrospective FDG-PET/CT analysis of the registered phase II trial NCT02919683, although a prospective analysis plan specifying the detailed imaging–pathological response analyses of the secondary report was not identified. For Wondergem et al. [9], the NeoNivo trial registration and protocol prospectively specified PD-L1 and FDG PET measurements, longitudinal uptake changes, and relationships between imaging, treatment response, and biological markers. Jiang et al. [10] described the FAPI-PET response analysis as a prespecified exploratory aim within the corresponding registered clinical protocols. No study-specific prospective radiomics-analysis protocol or registration was identified for Ma et al. [11] or Lin et al. [12]. Yuan et al. [13] reported a trial registration, but this was submitted after completion of the retrospective data collection period and therefore did not constitute prospective specification of the reported radiomics analysis. Ding et al. [14] linked its cohort to registered neoadjuvant chemoimmunotherapy trials, although a prospective registration specifically prespecifying the DeltaHabitat modeling analysis was not identified. No overt discrepancy in review-relevant imaging-response outcomes was identified where sufficiently detailed prospective documentation was available; however, selective outcome or analytical reporting could not be reliably excluded across the evidence base. Study-specific information on registration, protocol availability, and assessment of selective outcome or analytical reporting is summarized in Supplementary Table S7. Formal assessment of publication bias or small-study effects was not performed because the prespecified minimum of 10 sufficiently comparable independent reports contributing to a common quantitative synthesis was not met.

Confidence in the evidence varied according to the characteristics of the contributing reports and was constrained by the limited number of eligible studies, differences in imaging modality and acquisition timing, heterogeneity in treatment-response definitions and treatment settings, and the absence of independently reported external validation specifically in OSCC/OCSCC populations for the computational models. Additional limitations identified in the study-level assessments included concerns related to analytical procedures, limited sample sizes in several biomarker studies, and variable radiomics-specific methodological quality. Directness was reduced for Lin et al. [12] because the oral SCC estimate relevant to this review originated from a model developed in a broader HNSCC population. Potential partial participant overlap between Lin et al. [12] and Ding et al. [14] also reduced the independence of part of the MRI evidence. Across the three functional imaging groups, no imaging biomarker or computational model was independently replicated in a separate OSCC population using the same imaging construct, imaging time point, response definition, and validation framework.

#### Suitability for Quantitative Synthesis and Exploratory Meta-Analysis

The feasibility of exploratory quantitative pooling was assessed using the prespecified criteria of population, treatment setting, imaging modality or analytical class, imaging time point, response definition, performance metric, and cohort independence. No group of at least three independent reports fulfilled all of these criteria; therefore, no meta-analysis was performed.

The three PET-based reports [8,9,10] could not be quantitatively combined. Shah et al. [8] evaluated longitudinal FDG-PET/CT changes in relation to pathological response but did not report an AUC for the primary imaging-response association. Wondergem et al. [9] evaluated both FDG-PET and PD-L1-targeted PET and likewise did not provide a discrimination estimate suitable for pooling. Jiang et al. [10] evaluated serial ^68Ga-FAPI-04 PET/CT and reported AUCs for ΔSUV measures against MPR. Thus, the PET reports differed in tracer, quantitative biomarker, response definition, and available performance metric, and only one provided an AUC for a directly defined pathological response classification.

Ma et al. [11] was the only CT-radiomics report and evaluated pretreatment prediction of iRECIST-defined radiological response. Consequently, no additional CT-based study was available for quantitative synthesis of this outcome. The MRI-based reports of Lin et al. [12], Yuan et al. [13], and Ding et al. [14] all reported AUCs for pCR after NACI, but these estimates represented different prediction tasks and were therefore not considered quantitatively interchangeable. Lin et al. [12] evaluated pretreatment MRI radiomics, so its AUC reflected the ability to predict pCR before NACI began. Yuan et al. [13] derived habitat features from MRI obtained after NACI and shortly before surgery, so its AUC reflected post-treatment response assessment using information unavailable at baseline. Ding et al. [14] quantified DeltaHabitat changes between pre- and post-NACI MRI examinations, making its prediction dependent on longitudinal information from both time points. Thus, although the three studies shared MRI as the imaging modality and pCR as the outcome, their models used fundamentally different information sets available at different clinical decision points. Pooling their AUCs would therefore average discrimination across non-equivalent prediction tasks rather than estimate the performance of a common imaging strategy. In addition, Lin et al. [12] and Ding et al. [14] were classified as potentially non-independent because of possible partial participant overlap at Sun Yat-sen Memorial Hospital.

The available validation estimates also arose from different evaluation procedures. Ma et al. [11] used LOOCV, Lin et al. [12] reported an oral-subgroup estimate from a model developed and validated in a broader HNSCC population, Yuan et al. [13] reported performance from five-fold cross-validation, and Ding et al. [14] reported results from a same-center held-out test set. These estimates were retained separately in the structured synthesis rather than combined into a common pooled performance measure.

Similarly, a bivariate meta-analysis of sensitivity and specificity was not undertaken because at least three independent studies with comparable imaging approaches, response definitions, and reconstructable classification data at compatible thresholds were not available. Formal assessment of small-study effects using funnel plots or regression-based tests was also not performed because no quantitative synthesis contained the minimum prespecified number of 10 comparable independent studies.

Accordingly, the prespecified structured functional meta-synthesis constituted the final synthesis approach for the included evidence.

### 3.9. Structured Functional Meta-Synthesis

**Figure 3:**
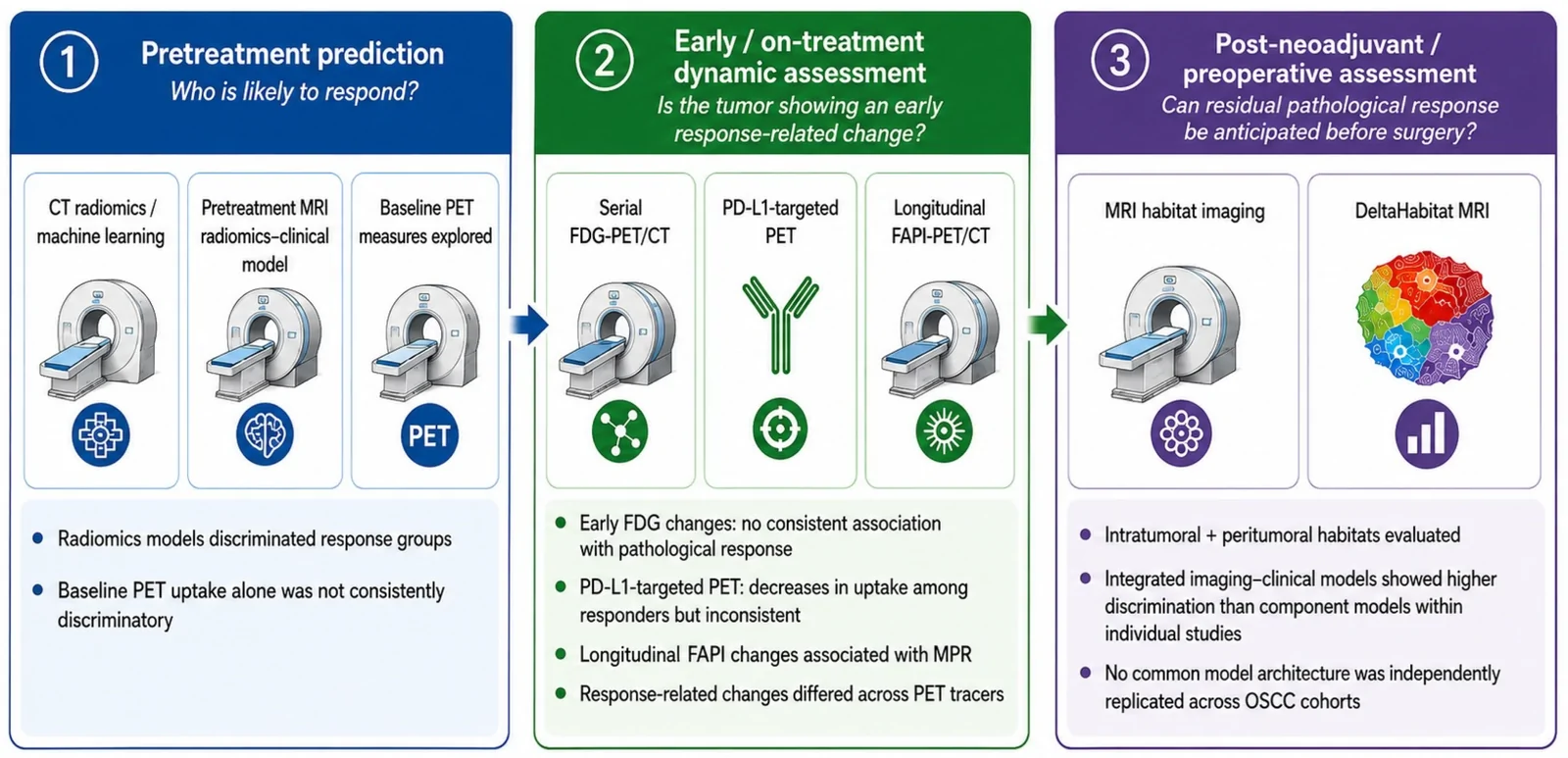
Structured functional meta-synthesis of imaging biomarkers according to their clinical role and timing in immunotherapy-response assessment.

The structured functional meta-synthesis organized the available evidence according to imaging timing and intended clinical role in relation to immunotherapy-based treatment. Three distinct groups were identified: pretreatment prediction, early/on-treatment dynamic assessment, and post-neoadjuvant/preoperative response assessment (Figure 3). Because baseline and longitudinal measurements within the same PET study addressed different clinical questions, individual reports could contribute information to more than one functional group.

Pretreatment prediction included CT and MRI radiomics models and baseline quantitative PET measurements. Ma et al. [11] evaluated pretreatment CT radiomics for iRECIST-defined response; the neural-network classifier provided the highest reported discrimination among the evaluated models (AUC 0.864). Lin et al. [12] evaluated a pretreatment MRI radiomics–clinical model for pCR after NACI and reported an AUC of 0.816 (95% CI, 0.725–0.906) for the oral SCC subgroup. In contrast, baseline molecular-imaging measurements did not consistently distinguish subsequent pathological response. Wondergem et al. [9] found no significant difference in baseline PD-L1-targeted PET SUVpeak between responders and non-responders, and Jiang et al. [10] found that baseline FAPI uptake was not significantly associated with MPR. Thus, within the available pretreatment evidence, discriminatory performance was reported for radiomics-based models, whereas single baseline PET uptake measures were not consistently discriminatory.

Early/on-treatment dynamic assessment was represented by the three longitudinal PET studies [8,9,10]. Shah et al. [8] found no significant association between early change in primary-tumor FDG SUVmax and subsequent pathological response. Wondergem et al. [9] likewise did not report a discriminatory AUC for serial PET, although FDG SUVpeak decreased after treatment in all patients who achieved MPR or partial pathological response. In contrast, Jiang et al. [10] reported that longitudinal FAPI changes discriminated MPR, with ΔSUVmax and ΔSUVpeak each yielding an AUC of 0.927 and ΔSUVmean an AUC of 0.917. Accordingly, the longitudinal PET evidence was not uniform: response-related changes differed according to the tracer and quantitative measure.

Post-neoadjuvant/preoperative assessment was represented by the two MRI studies incorporating spatial or longitudinal tumor-heterogeneity information. Yuan et al. [13] evaluated intratumoral and peritumoral habitat features derived from MRI obtained after NACI and before surgery; the integrated habitat–clinical model achieved a mean testing AUC of 0.843 across five-fold cross-validation. Ding et al. [14] quantified longitudinal changes in MRI-defined habitats between pre- and post-NACI examinations; the DeltaHabitat model achieved a test-set AUC of 0.878, increasing to 0.915 when combined with clinical variables. Within these individual studies, integrated imaging–clinical approaches showed higher reported discrimination than their respective imaging-only components.

Overall, the evidence therefore comprised three clinically distinct imaging-response frameworks rather than a single homogeneous prediction task. Pretreatment approaches estimated response susceptibility before therapy, longitudinal PET biomarkers characterized treatment-related change after therapy had begun, and post-neoadjuvant MRI models assessed response shortly before definitive pathological evaluation. However, no imaging biomarker or computational model was independently replicated across OSCC cohorts using the same imaging construct, treatment time point, response definition, and validation framework. These functional distinctions and the absence of independent replication formed the basis of the final structured synthesis.

The available evidence was organized into three functionally distinct groups according to imaging timing and intended clinical use: pretreatment prediction, early/on-treatment dynamic assessment, and post-neoadjuvant/preoperative response assessment. Pretreatment approaches included CT radiomics and machine-learning, MRI radiomics–clinical modeling, and baseline quantitative PET measurements. Early/on-treatment assessment comprised serial FDG-PET/CT, PD-L1-targeted PET, and longitudinal FAPI-PET/CT. Post-neoadjuvant/preoperative approaches incorporated MRI habitat imaging and longitudinal DeltaHabitat analysis. The three groups address different stages of response assessment and were therefore synthesized separately. (Abbreviations: CT, computed tomography; FAPI, fibroblast activation protein inhibitor; FDG, fluorodeoxyglucose; MPR, major pathological response; MRI, magnetic resonance imaging; OSCC, oral squamous cell carcinoma; PD-L1, programmed death ligand 1; PET/CT, positron emission tomography/computed tomography).

## 4. Discussion

This systematic review identifies three clinically distinct roles for computational imaging in immunotherapy-treated OSCC: pretreatment prediction, early/on-treatment dynamic assessment, and post-neoadjuvant/preoperative response assessment. This distinction has practical relevance as immunotherapy moves into potentially curable disease. Perioperative pembrolizumab has demonstrated clinical benefit in resectable locally advanced HNSCC [3], and pathological response after neoadjuvant immune checkpoint inhibition is associated with subsequent disease-free survival [28]. In this evolving setting, imaging could ultimately contribute at different decision points: pretreatment models could support baseline risk stratification, early dynamic biomarkers could identify treatment-related biological change, and post-neoadjuvant imaging could complement multidisciplinary assessment before surgery. These potential roles remain investigational, however, and none of the reviewed approaches currently provides evidence sufficient to alter treatment or surgical management.

The PET findings illustrate both the opportunities and the interpretive challenges of dynamic imaging. Conventional changes in FDG uptake were not consistently associated with pathological response in the eligible oral-cavity studies [8,9], whereas longitudinal FAPI-derived changes discriminated MPR in the small prospective cohort of Jiang et al. [10]. Evidence from the broader HNSCC literature is consistent with the possibility that more comprehensive longitudinal metabolic measures may outperform simple SUV-based assessment and also highlights the potential confounding effect of immune-related inflammatory uptake [29]. Thus, the clinically relevant signal may lie less in a single baseline or post-treatment uptake value than in appropriately selected longitudinal measures, although the encouraging FAPI findings require independent replication.

The radiomics studies represent a complementary pathway. Pretreatment CT and MRI models [11,12] sought to estimate response susceptibility before treatment, whereas habitat and DeltaHabitat approaches [13,14] used spatial or longitudinal information obtained after therapy had begun. Reported discrimination was encouraging within individual cohorts, and integrated imaging–clinical approaches sometimes outperformed their imaging-only components. However, higher AUCs from post-treatment or longitudinal models should not be interpreted as evidence of intrinsic superiority over pretreatment radiomics because these models have access to additional treatment-response information and address later clinical questions. This distinction also separates the present evidence from the broader MRI-radiomics literature in HNSCC, where recent quantitative synthesis has focused on neoadjuvant chemotherapy rather than immunotherapy and has included anatomically heterogeneous head and neck populations [15].

The reported biological associations provide a potential translational rationale for computational imaging beyond conventional morphology. Imaging features or molecular-imaging measures were associated with PD-L1-related variables, immune-cell characteristics, fibroblast-associated findings, or stromal immune-cell density in several included studies [9,10,12,13]. Their potential value is therefore more plausibly complementary to, rather than substitutive for, tissue biomarkers: imaging can characterize spatially extensive and longitudinal tumor phenotypes, whereas tissue analysis provides cellular and molecular specificity [30,31]. Lin et al. [12] compared an integrated radiomics–clinical model with CPS and component models and reported incremental predictive value, but oral-cavity-specific incremental benefit was not independently validated, and no included study demonstrated that imaging-guided decisions improved outcomes beyond established pathological information. Biological translation would require prespecified hypotheses, spatially and temporally matched imaging–tissue sampling, adequately powered cohorts, and independent replication of the same imaging–biological association across populations and centers. The currently reported associations therefore remain exploratory rather than validated mechanistic biomarkers.

The major constraint on translation was not discrimination alone but the way the models were developed and validated. No computational model included in this review had independently reported external validation specifically in a separate OSCC/OCSCC population. Lin et al. [12] provided external validation for the broader HNSCC model, but the oral-cavity-specific performance estimate remained a subgroup result, whereas the other computational studies relied on LOOCV, cross-validation, or same-center held-out testing. PROBAST+AI identified the analysis domain as the principal source of concern across all four prediction-model reports [22]. Recurring limitations included limited effective sample size relative to model complexity, incomplete evidence that feature-selection and preprocessing procedures were fully contained within resampling, and data-partitioning strategies that did not always provide a fully independent assessment of performance [32]. These limitations can promote overfitting and optimistic performance estimates and therefore limit confidence in transportability across institutions, scanners, acquisition protocols, segmentation practices, and patient populations [33]. METRICS likewise showed substantial variation in radiomics-specific methodological quality [24]. Calibration and decision-curve analysis were reported in some studies, but neither establishes clinical effectiveness. Before an imaging biomarker could influence neoadjuvant treatment, surgical timing, or perioperative management, independent multicenter validation, reliable calibration in the intended population, and prospective evidence that model-guided decisions improve patient-relevant outcomes would be required.

The decision not to perform a meta-analysis was therefore a methodological consequence of non-comparability rather than simply of the small number of eligible reports. The closest apparent cluster comprised the three MRI studies reporting pCR-related AUCs [12,13,14], but their estimates addressed different clinical tasks. Lin et al. [12] used pretreatment MRI, Yuan et al. [13] evaluated post-NACI/preoperative habitat imaging, and Ding et al. [14] modeled longitudinal change between pre- and post-NACI examinations. These time points determine what information is available to each model, so their AUCs cannot be regarded as repeated estimates of a common MRI-biomarker performance parameter. Comparability was further reduced by different radiomic representations and validation frameworks, together with possible partial participant overlap between Lin et al. [12] and Ding et al. [14]. Pooling these estimates could therefore generate a statistically precise summary without a coherent clinical interpretation. The PET evidence was even less amenable to pooling because of differences in tracer, quantitative measure, response endpoint, and available discrimination statistics. The SWiM-informed functional meta-synthesis consequently preserved these clinically meaningful distinctions rather than imposing a common effect measure on non-equivalent imaging tasks [25].

This review has several methodological strengths, including strict oral-cavity-specific eligibility, separation of imaging approaches according to intended clinical use, design-matched risk-of-bias assessment, complementary radiomics appraisal with METRICS, explicit assessment of cross-publication participant overlap, and consideration of available registrations and protocols. Important limitations remain. Only seven reports met the eligibility criteria; most computational evidence originated from China; several biomarker cohorts were small; response definitions varied; one eligible estimate came from an oral subgroup of a broader HNSCC model; and possible participant overlap affected part of the MRI evidence. Prospective analysis plans were also unavailable for several computational studies, preventing reliable exclusion of selective analytical reporting. Exclusion of conference abstracts improved methodological evaluability but may have omitted very recent preliminary evidence, and the rapid evolution of both immunotherapy regimens and imaging analytics means that the evidence base is likely to expand after the July 2026 search cutoff. Given the small and rapidly evolving evidence base, preferential publication of positive or high-performing models also cannot be excluded and could make the published literature appear more favorable than the underlying research record.

Future studies should prioritize prospective multicenter OSCC cohorts with prespecified clinical use and imaging time point. Shared acquisition, reconstruction, segmentation, and preprocessing protocols, collaborative multicenter consortia, and, where governance permits, de-identified imaging repositories or federated datasets could facilitate independent validation. Critical analytical priorities are to keep all data-driven preprocessing, feature selection, and model tuning within the training procedure, lock the final model before external testing, and report calibration alongside discrimination. Performance requirements should be decision-specific rather than based on a universal AUC or predictive-value threshold: pretreatment models would require high sensitivity and reliable calibration, early-response models prospectively validated change thresholds robust to treatment-related inflammatory effects, and models considered for surgical de-escalation extremely low tolerance for false-negative residual disease with prospective confirmation against surgical pathology. Implementation studies should also assess workflow integration, imaging and tracer availability, computational infrastructure, cost-effectiveness, and feasibility across different resource settings. Center-separated external validation should precede prospective clinical-impact studies demonstrating that model-guided decisions improve patient management.

## 5. Conclusions

Current evidence supports three functionally distinct roles for computational imaging in immunotherapy-treated OSCC: pretreatment prediction, early/on-treatment dynamic assessment, and post-neoadjuvant/preoperative response assessment. None is currently ready for routine clinical use. Among these approaches, post-neoadjuvant/preoperative MRI habitat and longitudinal DeltaHabitat modeling currently provide the most consistent signal of promise, because two studies reported meaningful discrimination of pathological complete response and showed improved performance when imaging information was integrated with clinical variables. However, these models use information obtained after treatment has begun and remain insufficiently validated, so their apparently higher discrimination should not be interpreted as evidence of superiority over pretreatment approaches. Longitudinal FAPI-PET also showed strong discrimination of major pathological response, but this finding derives from a single small cohort and requires independent replication.

Future studies should move beyond repeated single-cohort model development. The intended clinical decision and imaging time point should be prespecified; image acquisition, reconstruction, preprocessing, and segmentation procedures should be harmonized; feature definitions and extraction should follow standardized frameworks; and all data-driven preprocessing, feature selection, and model tuning should be confined to the training procedure to minimize information leakage and overfitting. Final models should be locked before evaluation in center-separated, independent OSCC cohorts, with calibration, uncertainty, incremental value over clinical predictors, and clinical utility reported alongside discrimination. Ultimately, prospective impact studies will be required to determine whether imaging-guided response assessment improves treatment selection, perioperative decision making, or patient outcomes before these biomarkers can support response-adapted immunotherapy strategies in OSCC.

## Figures and Tables

**Table 1:** Characteristics of included reports, study populations, and treatment settings.

Study	Country; Design and Recruitment Setting	Eligible Population	Sample Contributing to the Review	Treatment Context	Response Endpoint/Reference Standard
Shah et al. [8]	USA; retrospective imaging analysis of a prospective single-center phase II open-label randomized trial; 2016–2019	Previously untreated, surgically resectable OCSCC, clinical T2 or higher and/or clinically node-positive	29 enrolled; 27 evaluable for the reported response analysis	3 weeks of nivolumab or nivolumab plus ipilimumab, followed by surgery	Primary-tumor pathological response assessed from the surgical specimen
Wondergem et al. [9]	Netherlands; prospective single-center phase II study; March 2019–July 2021	Treatment-naïve stage III/IV OSCC	17 enrolled; 16 evaluable	Single 480 mg dose of nivolumab approximately 3 weeks before surgery	Pathological response after surgery, including MPR
Jiang et al. [10]	China; prospective exploratory biomarker study embedded in clinical protocols; November 2021–June 2024	Treatment-naïve, resectable locally advanced OSCC, stage III–IVA	31 enrolled; 20 included in the final analysis	Two cycles of NACI followed by radical surgery	MPR after surgery, including pCR
Ma et al. [11]	China; retrospective single-center study; April 2020–February 2023	Locally advanced or recurrent/metastatic OSCC receiving immunotherapy	40	PD-1 inhibitor-based immunotherapy; no local treatment during the treatment interval	Radiological immunotherapy response from follow-up imaging; responders comprised iCR/iPR and non-responders iSD/iCPD
Lin et al. [12]	China; retrospective three-center study; development/internal cohorts January 2021–February 2023 and external cohort April 2021–April 2024	Mixed HNSCC cohort with separately reported oral SCC subgroup	172 total HNSCC; 90 oral SCC patients relevant to this review	Two or three cycles of chemotherapy plus a PD-1 inhibitor, followed by radical surgery	pCR on postoperative pathological examination
Yuan et al. [13]	China; retrospective two-center study; March 2020–December 2023	OSCC treated with NACI and subsequent surgery	244 screened; 212 included	NACI followed by surgery	pCR on postoperative pathological assessment
Ding et al. [14]	China; retrospective single-center study; November 2020–April 2023	Non-metastatic OCSCC treated within neoadjuvant chemoimmunotherapy trials	540 identified; 195 included	Predominantly 2–4 cycles of PD-1 inhibitor plus nab-paclitaxel and platinum, followed by curative-intent surgery	pCR defined by absence of residual viable tumor in both primary tumor and lymph nodes

HNSCC, head and neck squamous cell carcinoma; iCPD, immune confirmed progressive disease; iCR, immune complete response; iPR, immune partial response; iSD, immune stable disease; MPR, major pathological response; NACI, neoadjuvant chemoimmunotherapy; OCSCC, oral cavity squamous cell carcinoma; OSCC, oral squamous cell carcinoma; pCR, pathological complete response; PD-1, programmed cell death protein 1; USA, United States of America.

**Table 2:** Imaging characteristics of quantitative PET biomarker studies.

Study	Imaging Modality and Timing	Image Region/Assessment	Quantitative Imaging Measures	Analytical Approach
Shah et al. [8]	FDG-PET/CT at baseline and after preoperative immunotherapy, before surgery	Primary tumor and cervical LNs; PET findings reviewed by nuclear medicine physicians	SUVmax; absolute and percentage change in SUVmax; CT-derived lesion size; newly FDG-avid LNs	Longitudinal semiquantitative imaging analysis; no radiomics or multivariable ML model
Wondergem et al. [9]	^18F-BMS-986192 PD-L1 PET and FDG PET at baseline and after nivolumab, before surgery	Primary-tumor tracer uptake on serial PET examinations	PD-L1 PET SUVpeak and TLA; FDG SUVpeak and TLG; longitudinal uptake changes	Dual-tracer longitudinal quantitative imaging; no radiomics or multivariable ML model
Jiang et al. [10]	^68Ga-FAPI-04 PET/CT at baseline and preoperatively after two NACI cycles	Lesion assessment by two nuclear medicine physicians; disagreements resolved by consensus	SUVmax, SUVmean, SUVpeak, and percentage change in serial SUV measures	Longitudinal semiquantitative molecular imaging; no radiomics or multivariable ML model

CT, computed tomography; FAPI, fibroblast activation protein inhibitor; FDG, fluorodeoxyglucose; LN, lymph node; NACI, neoadjuvant chemoimmunotherapy; PD-L1, programmed death ligand 1; PET, positron emission tomography; PET/CT, positron emission tomography/computed tomography; SUV, standardized uptake value; SUVmax, maximum standardized uptake value; SUVmean, mean standardized uptake value; SUVpeak, peak standardized uptake value; TLA, total lesion activity; TLG, total lesion glycolysis.

**Table 3:** Imaging and computational characteristics of radiomics and machine-learning studies.

Study	Imaging Modality and Timing	Segmentation/Spatial Representation	Feature Representation and Preprocessing	Feature Selection/Modeling
Ma et al. [11]	Pretreatment maxillofacial CT	Manual two-dimensional segmentation of the largest axial tumor section	Shape, first-order, and texture features from original, LoG-filtered, and wavelet-transformed images; z-score standardization	Univariate filtering and LASSO feature selection; NN, SVM, RF, and LR classifiers
Lin et al. [12]	Pretreatment multicenter MRI before NACI; T2WI and CE-T1WI	Whole tumor manually segmented; concentric peritumoral regions generated by morphological operations	Intratumoral and peritumoral handcrafted radiomic features; z-score normalization; reproducibility filtering; separate Rad-scores	LASSO feature selection; radiomic signatures and radiomics-clinical LR nomograms incorporating CPS
Yuan et al. [13]	Multisequence MRI after NACI and before surgery; T1WI, T2WI, and T1C	Whole-tumor segmentation with 3 mm peritumoral region; five K-means-defined habitats	N4 bias-field correction, isotropic resampling, intersequence registration; shape, first-order, and texture features within habitats	Correlation filtering and LASSO; LR, SVM, KNN, DT, and RF models
Ding et al. [14]	CE-T1WI before and after NACI; post-treatment MRI shortly before surgery	Whole primary tumor manually segmented; three K-means-defined habitats on serial examinations	Conventional radiomics, habitat features, and temporal changes including DeltaHabitat	Delta-radiomics, pre-habitat, DeltaHabitat, clinical, and combined models using LR, RF, ExtraTrees, XGBoost, and LightGBM

CE-T1WI, contrast-enhanced T1-weighted imaging; CPS, combined positive score; CT, computed tomography; DT, decision tree; KNN, k-nearest neighbors; LASSO, least absolute shrinkage and selection operator; LightGBM, Light Gradient Boosting Machine; LoG, Laplacian of Gaussian; LR, logistic regression; MRI, magnetic resonance imaging; NACI, neoadjuvant chemoimmunotherapy; NN, neural network; RF, random forest; SVM, support vector machine; T1C, contrast-enhanced T1-weighted imaging with fat suppression; T1WI, T1-weighted imaging; T2WI, T2-weighted imaging; XGBoost, eXtreme Gradient Boosting.

**Table 4:** Treatment-response outcomes and predictive performance of the included reports.

Study	Response Endpoint	Response Distribution	Principal Imaging Biomarker/Model	Main Treatment-Response Finding and Performance
Shah et al. [8]	Continuous pathological response in the primary tumor after neoadjuvant immunotherapy	27 patients with evaluable serial FDG-PET/CT	Change in primary-tumor FDG SUVmax	No correlation between early change in SUVmax and subsequent pathological response; no AUC reported
Wondergem et al. [9]	Pathological response, including MPR	6/16 had a pathological response; 3/16 achieved MPR	Serial PD-L1-targeted PET and FDG-PET uptake	Baseline PD-L1 PET SUVpeak did not significantly differ between responders and non-responders (median 5.3 vs. 3.4; *p* = 0.54). FDG SUVpeak decreased in all patients with MPR or partial pathological response; no AUC reported
Jiang et al. [10]	MPR versus non-MPR; pCR included within MPR	8/20 MPR, including 6 pCR; 12/20 non-MPR	Longitudinal ^68Ga-FAPI-04 PET/CT ΔSUV measures	ΔSUVmax: AUC 0.927 (95% CI, 0.816–1.000), sensitivity 75.0%, specificity 100.0%; ΔSUVmean: AUC 0.917 (95% CI, 0.796–1.000); ΔSUVpeak: AUC 0.927 (95% CI, 0.816–1.000)
Ma et al. [11]	iRECIST-defined radiological response: iCR/iPR versus iSD/iCPD	23 responders; 17 non-responders	Pretreatment CT-radiomics neural-network model	AUC 0.864; accuracy 0.825; sensitivity 0.825; specificity 0.825. Alternative models: SVM 0.813, RF 0.777, LR 0.767
Lin et al. [12]	pCR versus non-pCR after NACI	Oral SCC subgroup, n = 90	Pretreatment MRI radiomics–clinical nomogram	Oral-specific AUC 0.816 (95% CI, 0.725–0.906)
Yuan et al. [13]	pCR versus non-pCR after NACI	56/212 pCR; 156/212 non-pCR	Integrated intratumoral/peritumoral habitat–clinical decision model	Mean AUC 0.913 in training and 0.843 in testing; mean test accuracy 0.788 across five-fold cross-validation
Ding et al. [14]	pCR versus non-pCR after NACI	59/195 pCR; 136/195 non-pCR	DeltaHabitat model and combined DeltaHabitat–clinical model	DeltaHabitat: AUC 0.923 (95% CI, 0.880–0.967) training and 0.878 (95% CI, 0.791–0.964) test. Combined model: AUC 0.964 (95% CI, 0.936–0.991) training and 0.915 (95% CI, 0.841–0.988) test

AUC, area under the receiver operating characteristic curve; CI, confidence interval; FDG, fluorodeoxyglucose; iCPD, immune confirmed progressive disease; iCR, immune complete response; iPR, immune partial response; iRECIST, immune Response Evaluation Criteria in Solid Tumors; iSD, immune stable disease; LOOCV, leave-one-out cross-validation; LR, logistic regression; MPR, major pathological response; NACI, neoadjuvant chemoimmunotherapy; pCR, pathological complete response; PD-L1, programmed death ligand 1; PET/CT, positron emission tomography/computed tomography; RF, random forest; SCC, squamous cell carcinoma; SUVmax, maximum standardized uptake value; SUVpeak, peak standardized uptake value; SVM, support vector machine; ΔSUV, percentage change in standardized uptake value.

**Table 5:** Validation, calibration, clinical utility, and interpretability of computational imaging models.

Study	Validation Framework	Calibration	Clinical Utility and Incremental Value	Interpretability and Reproducibility
Ma et al. [11]	LOOCV within the original 40-patient cohort; no independent test or external validation	Not reported	DCA showed positive net benefit for all four models relative to treat-all/treat-none strategies at threshold probabilities of approximately 0.20–0.60	No dedicated explainability method reported. ROI delineation by one radiologist with senior-radiologist confirmation; quantitative segmentation reproducibility not reported
Lin et al. [12]	Separate training, internal-validation, and external-validation cohorts across three centers. External validation applied to the overall HNSCC model; external performance not reported separately for oral SCC	Calibration curves reported for the radiomics–clinical nomogram	DCA showed net benefit over treat-all/treat-none strategies across the examined thresholds. Model compared with CPS, clinical nomogram, and component Rad-scores; incremental predictive value was reported	Histopathological interpretability analysis in 38 pretreatment biopsies using digital quantification of tumor-microenvironment components. Pathological measurements incorporated interobserver ICC procedures
Yuan et al. [13]	Five-fold cross-validation with repeated training/validation folds; no independent center-separated external validation	Calibration curves reported for the decision model in the training and testing sets	Formal DCA or net-benefit analysis not reported	SHAP-based feature attribution used to identify influential intratumoral and peritumoral habitat features. Manual tumor segmentation reviewed by a second experienced radiologist; quantitative segmentation reproducibility not reported
Ding et al. [14]	Single-center 7:3 training/test split; no external validation	Calibration curves and Hosmer–Lemeshow test	DCA performed for models in training and held-out test cohorts; combined imaging–clinical model evaluated against component models	No dedicated post hoc explainability framework reported. Longitudinal subvolume registration identified as a potential source of feature instability and bias

CPS, combined positive score; DCA, decision curve analysis; HNSCC, head and neck squamous cell carcinoma; ICC, intraclass correlation coefficient; LOOCV, leave-one-out cross-validation; OSCC, oral squamous cell carcinoma; ROI, region of interest; Rad-score, radiomics score; SCC, squamous cell carcinoma; SHAP, SHapley Additive exPlanations.

## Data Availability

The original contributions presented in this study are included in the article/Supplementary Material. Further inquiries can be directed to the corresponding author.

## Supplementary Materials

The following supporting information can be downloaded at: https://www.mdpi.com/article/10.3390/medsci14050554/s1, Table S1. Search strategies used for database searches. Table S2. Excluded Full-Text Studies. Table S3. Standardized data extraction form used in the systematic review. Table S4. Cross Publication Overlap Matrix. Table S5. PROBAST AI QUIPS Assessment. Table S6. METRICS Assessment. Table S7. Prospective registration, protocol availability, and reporting-bias assessment of the included reports; PRISMA 2020.
