# Supplementary material for: Computational and AI-Enabled Imaging Biomarkers for Predicting and Assessing Immunotherapy Response in Oral Squamous Cell Carcinoma: A Systematic Review with Functional Meta-Synthesis
Source: Med Sci (Basel). 2026 Sep 8;14(5):554. doi: 10.3390/medsci14050554 (PMC13609177; doi:10.3390/medsci14050554)
Supplement: Supplementary file 1 [file medsci-14-00554-s001.zip › Supplementary Table S1 Search Strategies.pdf]

Supplementary Table S1. Complete search strategies

Database / source	Final consolidated search strategy	Limits / procedure	Records retrieved (n)
PubMed/MEDLINE	(<br>(<br>(<br>"Mouth Neoplasms"[Mesh]<br>OR "oral squamous cell carcinoma"[tiab]<br>OR "oral cavity squamous cell carcinoma"[tiab]<br>OR "oral tongue squamous cell carcinoma"[tiab]<br>OR OSCC[tiab]<br>OR OCSCC[tiab]<br>)<br>AND<br>(<br>"Radiomics"[Mesh]<br>OR radiomic*[tiab]<br>OR radiogenom*[tiab]<br>OR "artificial intelligence"[tiab]<br>OR "machine learning"[tiab]<br>OR "deep learning"[tiab]<br>OR "neural network*"[tiab]<br>OR "habitat radiomic*"[tiab]<br>OR "delta radiomic*"[tiab]<br>OR "quantitative imaging"[tiab]<br>OR PET[tiab]<br>OR "PET/CT"[tiab]<br>OR "positron emission tomography"[tiab]<br>OR "diffusion-weighted imaging"[tiab]<br>OR DWI[tiab]<br>OR "magnetic resonance imaging"[tiab]<br>)<br>AND<br>(<br>"Immunotherapy"[Mesh]<br>OR immunotherap*[tiab]<br>OR chemoimmunotherap*[tiab]<br>OR immunochemotherap*[tiab]<br>OR "immune checkpoint inhibitor*"[tiab]<br>OR "immune checkpoint blockade"[tiab]<br>OR PD-1[tiab]<br>OR PD-L1[tiab]<br>OR pembrolizumab[tiab]<br>OR nivolumab[tiab]<br>OR ipilimumab[tiab]<br>OR camrelizumab[tiab]<br>OR sintilimab[tiab]<br>OR toripalimab[tiab]<br>OR tislelizumab[tiab]<br>OR durvalumab[tiab]<br>OR tremelimumab[tiab]<br>)<br>)<br>OR<br>(<br>(	From inception to July 31 2026; no language/date restrictions.	154

Database / source	Final consolidated search strategy	Limits / procedure	Records retrieved (n)
	"Squamous Cell Carcinoma of Head and Neck"[Mesh]<br>OR "head and neck squamous cell carcinoma"[tiab]<br>OR HNSCC[tiab]<br>)<br>AND<br>(<br>radiomic*[tiab]<br>OR radiogenom*[tiab]<br>OR "artificial intelligence"[tiab]<br>OR "machine learning"[tiab]<br>OR "deep learning"[tiab]<br>OR "neural network"[tiab]<br>OR "neural networks"[tiab]<br>OR "habitat radiomics"[tiab]<br>OR "delta radiomics"[tiab]<br>OR "delta habitat"[tiab]<br>OR "quantitative imaging"[tiab]<br>)<br>AND<br>(<br>immunotherap*[tiab]<br>OR chemoimmunotherap*[tiab]<br>OR immunochemotherap*[tiab]<br>OR "immune checkpoint inhibitor"[tiab]<br>OR "immune checkpoint inhibitors"[tiab]<br>OR "immune checkpoint blockade"[tiab]<br>OR PD-1[tiab]<br>OR PD-L1[tiab]<br>OR pembrolizumab[tiab]<br>OR nivolumab[tiab]<br>OR ipilimumab[tiab]<br>OR camrelizumab[tiab]<br>OR sintilimab[tiab]<br>OR toripalimab[tiab]<br>OR tislelizumab[tiab]<br>OR durvalumab[tiab]<br>OR tremelimumab[tiab]<br>)<br>AND<br>(<br>predict*[tiab]<br>OR respons*[tiab]<br>OR "pathologic response"[tiab]<br>OR "pathological response"[tiab]<br>OR "complete response"[tiab]<br>OR "major pathological response"[tiab]<br>OR "major pathologic response"[tiab]<br>OR pCR[tiab]<br>OR MPR[tiab]<br>)<br>)<br>)		
Scopus	TITLE-ABS-KEY(<br>(<br>(	From inception to July 31 2026; no language/date restrictions.	286

Database / source	Final consolidated search strategy	Limits / procedure	Records retrieved (n)
	"oral squamous cell carcinoma"<br>OR "oral cavity squamous cell carcinoma"<br>OR "oral tongue squamous cell carcinoma"<br>OR OSCC<br>OR OCSCC<br>)<br>AND<br>(<br>radiomic*<br>OR radiogenom*<br>OR "artificial intelligence"<br>OR "machine learning"<br>OR "deep learning"<br>OR "neural network*"<br>OR "habitat radiomic*"<br>OR "delta radiomic*"<br>OR "quantitative imaging"<br>OR PET<br>OR "PET/CT"<br>OR "positron emission tomography"<br>OR "diffusion-weighted imaging"<br>OR DWI<br>)<br>AND<br>(<br>immunotherap*<br>OR chemoimmunotherap*<br>OR immunochemotherap*<br>OR "immune checkpoint inhibitor*"<br>OR "immune checkpoint blockade"<br>OR "PD-1"<br>OR "PD-L1"<br>OR pembrolizumab<br>OR nivolumab<br>OR ipilimumab<br>OR camrelizumab<br>OR sintilimab<br>OR toripalimab<br>OR tislelizumab<br>OR durvalumab<br>OR tremelimumab<br>)<br>)<br>OR<br>(<br>(<br>"head and neck squamous cell carcinoma"<br>OR HNSCC<br>)<br>AND<br>(<br>radiomic*<br>OR radiogenom*<br>OR "artificial intelligence"<br>OR "machine learning"		

Database / source	Final consolidated search strategy	Limits / procedure	Records retrieved (n)
	OR "deep learning"<br>OR "neural network*"<br>OR "habitat radiomics"<br>OR "delta radiomics"<br>OR "delta habitat"<br>OR "quantitative imaging"<br>)<br>AND<br>(<br>immunotherap*<br>OR chemoimmunotherap*<br>OR immunochemotherap*<br>OR "immune checkpoint inhibitor*"<br>OR "immune checkpoint blockade"<br>OR "PD-1"<br>OR "PD-L1"<br>OR pembrolizumab<br>OR nivolumab<br>OR ipilimumab<br>OR camrelizumab<br>OR sintilimab<br>OR toripalimab<br>OR tislelizumab<br>OR durvalumab<br>OR tremelimumab<br>)<br>AND<br>(<br>predict*<br>OR respons*<br>OR "pathologic response"<br>OR "pathological response"<br>OR "complete response"<br>OR "major pathological response"<br>OR "major pathologic response"<br>OR pCR<br>OR MPR<br>)<br>)<br>)		
Embase	(<br>(<br>(<br>'oral squamous cell carcinoma':ti,ab,kw<br>OR 'oral cavity squamous cell carcinoma':ti,ab,kw<br>OR 'oral tongue squamous cell carcinoma':ti,ab,kw<br>OR oscc:ti,ab,kw<br>OR ocscc:ti,ab,kw<br>)<br>AND<br>(<br>(<br>radiomic*:ti,ab,kw<br>OR radiogenom*:ti,ab,kw<br>OR 'artificial intelligence':ti,ab,kw	From inception to July 31 2026; no language/date restrictions.	213

Database / source	Final consolidated search strategy	Limits / procedure	Records retrieved (n)
	OR 'machine learning':ti,ab,kw<br>OR 'deep learning':ti,ab,kw<br>OR 'neural network':ti,ab,kw<br>OR 'neural networks':ti,ab,kw<br>OR 'habitat radiomics':ti,ab,kw<br>OR 'habitat radiomic':ti,ab,kw<br>OR 'delta radiomics':ti,ab,kw<br>OR 'delta radiomic':ti,ab,kw<br>OR 'delta habitat':ti,ab,kw<br>OR 'quantitative imaging':ti,ab,kw<br>)<br>OR<br>(<br>(<br>pet:ti,ab,kw<br>OR 'pet/ct':ti,ab,kw<br>OR 'positron emission tomography':ti,ab,kw<br>OR mri:ti,ab,kw<br>OR 'magnetic resonance imaging':ti,ab,kw<br>OR dwi:ti,ab,kw<br>OR 'diffusion weighted imaging':ti,ab,kw<br>OR 'computed tomography':ti,ab,kw<br>)<br>AND<br>(<br>predict*:ti,ab,kw<br>OR respons*:ti,ab,kw<br>OR biomarker*:ti,ab,kw<br>OR quantitative:ti,ab,kw<br>OR feature*:ti,ab,kw<br>)<br>)<br>)<br>AND<br>(<br>immunotherap*:ti,ab,kw<br>OR chemoimmunotherap*:ti,ab,kw<br>OR immunochemotherap*:ti,ab,kw<br>OR 'immune checkpoint inhibitor':ti,ab,kw<br>OR 'immune checkpoint inhibitors':ti,ab,kw<br>OR 'immune checkpoint blockade':ti,ab,kw<br>OR 'pd-1':ti,ab,kw<br>OR 'pd-l1':ti,ab,kw<br>OR pembrolizumab:ti,ab,kw<br>OR nivolumab:ti,ab,kw<br>OR ipilimumab:ti,ab,kw<br>OR camrelizumab:ti,ab,kw<br>OR sintilimab:ti,ab,kw<br>OR toripalimab:ti,ab,kw<br>OR tislelizumab:ti,ab,kw<br>OR durvalumab:ti,ab,kw<br>OR tremelimumab:ti,ab,kw<br>)<br>)<br>OR		

Database / source	Final consolidated search strategy	Limits / procedure	Records retrieved (n)
	(<br>(<br>'head and neck squamous cell carcinoma':ti,ab,kw<br>OR hnscc:ti,ab,kw<br>)<br>AND<br>(<br>radiomic*:ti,ab,kw<br>OR radiogenom*:ti,ab,kw<br>OR 'artificial intelligence':ti,ab,kw<br>OR 'machine learning':ti,ab,kw<br>OR 'deep learning':ti,ab,kw<br>OR 'neural network':ti,ab,kw<br>OR 'neural networks':ti,ab,kw<br>OR 'habitat radiomics':ti,ab,kw<br>OR 'delta radiomics':ti,ab,kw<br>OR 'delta habitat':ti,ab,kw<br>OR 'quantitative imaging':ti,ab,kw<br>)<br>AND<br>(<br>immunotherap*:ti,ab,kw<br>OR chemoimmunotherap*:ti,ab,kw<br>OR immunochemotherap*:ti,ab,kw<br>OR 'immune checkpoint inhibitor':ti,ab,kw<br>OR 'immune checkpoint inhibitors':ti,ab,kw<br>OR 'immune checkpoint blockade':ti,ab,kw<br>OR 'pd-1':ti,ab,kw<br>OR 'pd-l1':ti,ab,kw<br>OR pembrolizumab:ti,ab,kw<br>OR nivolumab:ti,ab,kw<br>OR ipilimumab:ti,ab,kw<br>OR camrelizumab:ti,ab,kw<br>OR sintilimab:ti,ab,kw<br>OR toripalimab:ti,ab,kw<br>OR tislelizumab:ti,ab,kw<br>OR durvalumab:ti,ab,kw<br>OR tremelimumab:ti,ab,kw<br>)<br>AND<br>(<br>predict*:ti,ab,kw<br>OR respons*:ti,ab,kw<br>OR 'pathologic response':ti,ab,kw<br>OR 'pathological response':ti,ab,kw<br>OR 'complete response':ti,ab,kw<br>OR 'major pathological response':ti,ab,kw<br>OR 'major pathologic response':ti,ab,kw<br>OR pcr:ti,ab,kw<br>OR mpr:ti,ab,kw<br>)<br>)<br>)		

Database / source	Final consolidated search strategy	Limits / procedure	Records retrieved (n)
Google Scholar	("oral squamous cell carcinoma" OR "oral cavity squamous cell carcinoma" OR "head and neck squamous cell carcinoma")<br>(radiomics OR "artificial intelligence" OR "machine learning" OR "deep learning" OR "quantitative imaging" OR PET OR "PET/CT" OR MRI OR "diffusion weighted imaging")<br>(immunotherapy OR chemoimmunotherapy OR "immune checkpoint inhibitor" OR "immune checkpoint blockade" OR "PD-1" OR "PD-L1")<br>(response OR "pathologic response" OR "pathological response" OR pCR OR MPR)	Supplementary source. First 200 results ranked by relevance screened.	200 screened

Each bibliographic database was represented by one consolidated search constructed from the exact search components developed during feasibility testing. Mixed HNSCC studies were eligible only when oral-cavity-specific response or model-performance data were separately extractable. The final consolidated searches were conducted in PubMed/MEDLINE, Scopus, and Embase, and the database-specific yields are reported in the final column. Google Scholar was used as a supplementary source, and the first 200 results ranked by relevance were screened.
