# Supplementary material for: Computational and AI-Enabled Imaging Biomarkers for Predicting and Assessing Immunotherapy Response in Oral Squamous Cell Carcinoma: A Systematic Review with Functional Meta-Synthesis
Source: Med Sci (Basel). 2026 Sep 8;14(5):554. doi: 10.3390/medsci14050554 (PMC13609177; doi:10.3390/medsci14050554)
Supplement: Supplementary file 1 [file medsci-14-00554-s001.zip › Supplementary Table S2 Excluded Full Text Studies.pdf]

Supplementary Table S2. Full-text reports excluded from the systematic review and reasons for exclusion

No.	Reference	Reason for exclusion
1	Hellwig K, Ellmann S, Eckstein M, Wiesmueller M, Rutzner S, Semrau S, et al. Predictive value of multiparametric MRI for response to single-cycle induction chemo-immunotherapy in locally advanced head and neck squamous cell carcinoma. Front Oncol. 2021;11:734872. doi:10.3389/fonc.2021.734872.	No eligible oral-cavity primary tumors in the analyzed cohort. Although oral cavity was permitted by the study eligibility criteria, the 22 patients included in the imaging analysis had tumors of the oropharynx, hypopharynx, supraglottic larynx, or multilevel head-and-neck sites; therefore, no eligible OSCC/OCSCC population was available for extraction.
2	van der Hulst HJ, Vos JL, Tissier R, Smit LA, Martens RM, Beets-Tan RGH, et al. Quantitative diffusion-weighted imaging analyses to predict response to neoadjuvant immunotherapy in patients with locally advanced head and neck carcinoma. Cancers (Basel). 2022;14(24):6235. doi:10.3390/cancers14246235.	Mixed HNSCC cohort without separately extractable oral-cavity-specific imaging-response estimates. Oral-cavity tumors comprised 21/24 evaluable patients, but the reported DWI/radiomic associations with pathological response were calculated for the mixed 24-patient HNSCC cohort, including three oropharyngeal cancers, without oral-cavity-only performance estimates.
3	Zandberg DP, Zenkin S, Ak M, Mamindla P, Peddagangireddy V, Hsieh R, et al. Evaluation of radiomics as a predictor of efficacy and the tumor immune microenvironment in anti-PD-1 mAb treated recurrent/metastatic squamous cell carcinoma of the head and neck patients. Head Neck. 2025;47(1):129-138. doi:10.1002/hed.27878.	Mixed HNSCC cohort without separately extractable oral-cavity-specific model performance. Oral cavity was the primary site in 22/61 patients (36%), but radiomic models for disease control, progression-free survival, and overall survival were reported for the entire recurrent/metastatic HNSCC cohort rather than the oral-cavity subgroup.
4	Ding C, Kang Y, Bai F, Bai G, Xian J. Development and validation of MRI-derived deep learning score for non-invasive prediction of PD-L1 expression and prognostic stratification in head and neck squamous cell carcinoma. Cancer Imaging. 2025;25(1):14. doi:10.1186/s40644-025-00837-5.	Mixed HNSCC cohort without separately extractable oral-cavity-specific immunotherapy-response performance. The MRI-derived deep learning score was primarily developed for PD-L1 prediction and was subsequently evaluated for durable clinical benefit in an ICI-treated HNSCC cohort; the reported treatment-response performance was not provided separately for oral-cavity tumors.
5	Chen X, Liu R, Zhou Y, Xiao Y, Feng C, Chen L, et al. Noninvasive characterization of tumor heterogeneity in HNSCC: from clinical utility to biological correlates. Adv Sci (Weinh). 2026:e75780. doi:10.1002/advs.75780.	Mixed HNSCC immunotherapy cohort without separately extractable oral-cavity-specific response/model-performance data. The imaging-derived intratumoral heterogeneity score was evaluated for pathological complete response in a 100-patient neoadjuvant immunotherapy set, but the response-prediction results were reported for HNSCC as a whole rather than specifically for oral-cavity tumors.
6	Lan T, Tan Y, Shi H, Kuang S, Zhang Y, Wang L, et al. Deep learning enhanced MRI radiomics in predicting pathological response of head and neck squamous carcinoma to neoadjuvant chemoimmunotherapy: a retrospective analysis. Int J Surg. 2026;112(2):3905-3915. doi:10.1097/JS9.0000000000003710.	Predominantly oral but mixed HNSCC cohort without separately reported oral-cavity-specific model performance. The 282-patient cohort included oral-cavity subsites as well as 11 oropharyngeal tumors, while training, testing, and external-validation performance estimates were reported for the complete mixed cohort; an oral-cavity-only analysis was not available.

Notes. Full-text exclusion was based on the prespecified requirement that mixed HNSCC studies provide separately extractable oral-cavity-specific treatment-response or model-performance data. HNSCC, head and neck squamous cell carcinoma; ICI, immune checkpoint inhibitor; OCSCC, oral cavity squamous cell carcinoma; OSCC, oral squamous cell carcinoma. References are formatted in Vancouver style; for articles with more than six authors, the first six authors are followed by “et al.”
