# Supplementary material for: Computational and AI-Enabled Imaging Biomarkers for Predicting and Assessing Immunotherapy Response in Oral Squamous Cell Carcinoma: A Systematic Review with Functional Meta-Synthesis
Source: Med Sci (Basel). 2026 Sep 8;14(5):554. doi: 10.3390/medsci14050554 (PMC13609177; doi:10.3390/medsci14050554)
Supplement: Supplementary file 1 [file medsci-14-00554-s001.zip › Supplementary Table S3 Standardized data extraction form used in the systematic review.pdf]

Supplementary Table S3. Standardized data extraction form used in the systematic review

Domain	Data items extracted
Study identification	Author; year; country; study design; prospective/retrospective status; recruiting institutions; recruitment period; trial registration
Population	Sample size; disease setting; oral subsite; tumor stage; eligibility criteria
Treatment	Immunotherapy regimen; chemotherapy components when applicable; number of cycles; surgical context
Imaging acquisition	Modality; scanner characteristics; sequence/tracer; imaging time point relative to treatment and surgery
Image analysis	Segmentation method; region/volume of interest; preprocessing; software
Quantitative imaging/radiomics	Quantitative imaging parameters; feature families; feature-selection procedures; habitat/delta-feature derivation
Model development	Candidate predictors; final feature set; model/algorithm; hyperparameter tuning; missing-data handling; class imbalance
Validation	Training/test split; cross-validation/bootstrap; internal validation; held-out testing; external validation
Performance	AUC/discrimination measures; sensitivity; specificity; accuracy; uncertainty estimates
Calibration and clinical utility	Calibration assessment; decision-curve analysis; incremental value
Interpretability/reproducibility	Interpretability methods; segmentation/feature reproducibility
Outcomes	Response definition; timing; reference standard; responders/non-responders
Additional items	Funding; conflicts of interest; protocol availability; biological correlates; potential cross-publication participant overlap
