# Supplementary material for: Computational and AI-Enabled Imaging Biomarkers for Predicting and Assessing Immunotherapy Response in Oral Squamous Cell Carcinoma: A Systematic Review with Functional Meta-Synthesis
Source: Med Sci (Basel). 2026 Sep 8;14(5):554. doi: 10.3390/medsci14050554 (PMC13609177; doi:10.3390/medsci14050554)
Supplement: Supplementary file 1 [file medsci-14-00554-s001.zip › Supplementary Table S4 Cross Publication Overlap Matrix.pdf]

Supplementary Table S4. Cross-publication participant-overlap assessment among included reports

Pairwise overlap judgments were based on recruitment institutions, recruitment periods, trial identifiers, eligibility criteria, treatment regimens, imaging protocols, authorship, and reported cohort characteristics.

Report	Shah et al. [8]	Wondergem et al. [9]	Ma et al. [11]	Lin et al. [12]	Jiang et al. [10]	Yuan et al. [13]	Ding et al. [14]
Shah et al. [8]	—	N	N	N	N	N	N
Wondergem et al. [9]	N	—	N	N	N	N	N
Ma et al. [11]	N	N	—	N	N	N	N
Lin et al. [12]	N	N	N	—	N	N	**P**
Jiang et al. [10]	N	N	N	N	—	N	N
Yuan et al. [13]	N	N	N	N	N	—	N
Ding et al. [14]	N	N	N	**P**	N	N	—

Abbreviations and symbols: N, no evidence of participant overlap identified from the published information; P, potential partial participant overlap; OCSCC, oral cavity squamous cell carcinoma; pCR, pathological complete response; SCC, squamous cell carcinoma; —, same report/not applicable.

Overlap note for Lin et al. [12] and Ding et al. [14]: Both reports included patients treated at Sun Yat-sen Memorial Hospital during substantially overlapping recruitment periods and evaluated pCR after neoadjuvant chemoimmunotherapy. Lin et al. [12] reported 90 oral SCC cases; 61 belonged to the Sun Yat-sen Memorial training/internal-validation cohorts and could therefore potentially overlap with the 195-patient single-center OCSCC cohort reported by Ding et al. [14]. The remaining 29 oral SCC cases in Lin et al. [12] were recruited from external centers and could not overlap with the Ding et al. [14] cohort. Individual-level linkage information was unavailable and the extent of actual overlap could not be established. Definitive assessment would require sufficiently detailed center-specific enrollment information allowing participants to be linked across publications, such as de-identified participant identifiers or equivalent linkage variables, together with precise recruitment dates and source-trial or cohort identifiers.

Interpretation: The seven eligible reports were retained because they contributed distinct eligible imaging analyses. Potentially overlapping reports were treated as non-independent evidence and were not permitted to contribute simultaneously to the same pooled participant denominator or exploratory meta-analysis.
