# Supplementary material for: Computational and AI-Enabled Imaging Biomarkers for Predicting and Assessing Immunotherapy Response in Oral Squamous Cell Carcinoma: A Systematic Review with Functional Meta-Synthesis
Source: Med Sci (Basel). 2026 Sep 8;14(5):554. doi: 10.3390/medsci14050554 (PMC13609177; doi:10.3390/medsci14050554)
Supplement: Supplementary file 1 [file medsci-14-00554-s001.zip › Supplementary Table S5 PROBAST AI QUIPS Assessment.pdf]

Supplementary Table S5. Detailed risk-of-bias, model-development quality, and model-evaluation assessment of the included reports

Panel A. QUIPS assessment of studies evaluating individual quantitative imaging biomarkers [23].

Report	Study participation	Study attrition	Predictor measurement	Outcome measurement	Study confounding	Statistical analysis and reporting
**Shah et al. [8]**	Low	Low	Moderate	Low	Moderate	Moderate
**Wondergem et al. [9]**	Low	Moderate	Low	Low	Moderate	High
**Jiang et al. [10]**	Low	High	Low	Low	Moderate	High

QUIPS domain rationale: Shah et al. [8] included a near-complete evaluable cohort and blinded pathological assessment, but baseline PET acquisition was not fully standardized because five examinations were performed at outside institutions; analyses were exploratory in a small cohort receiving two neoadjuvant regimens. Wondergem et al. [9] had limited attrition, but only three patients achieved MPR and multiple exploratory imaging and immune variables were examined. Jiang et al. [10] excluded 11 of 31 enrolled patients from the final response analysis and derived ROC thresholds and discrimination estimates in the same 20-patient cohort without independent validation.

QUIPS does not require a numerical summary score; domain-level judgments were therefore retained as the primary assessment.

Panel B. PROBAST+AI model-development quality assessment [22].

Domain	Signaling question	Ma et al. [11]	Lin et al. [12]	Yuan et al. [13]	Ding et al. [14]
**D1**	1.1 Appropriate data sources?	Y	Y	Y	Y
**D1**	1.2 Appropriate study design?	Y	Y	Y	Y
**D1**	1.3 Inclusion/exclusion yielded a representative dataset?	Y	PY	PY	PY
**D2**	2.1 Predictors defined and assessed similarly for all participants?	Y	Y	Y	Y
**D2**	2.2 Predictor preprocessing applied consistently?	Y	Y	Y	Y
**D2**	2.3 Predictor assessment performed without knowledge of the outcome?	Y	NI	NI	Y
**D2**	2.4 Predictors available at the intended time of model use?	Y	Y	Y	Y
**D3**	3.1 Outcome defined and assessed appropriately?	Y	Y	Y	Y
**D3**	3.2 Outcome defined and assessed similarly for all participants?	Y	Y	Y	Y
**D3**	3.3 Outcome assessed without knowledge/use of predictors?	PY	Y	PY	Y
**D3**	3.4 Predictor-outcome interval appropriate?	Y	Y	PY	Y
**D4**	4.1 Evidence/sample size reasonable for model development?	N	PN	PN	PN
**D4**	4.2 Continuous/categorical predictors handled appropriately?	Y	Y	Y	Y
**D4**	4.3 Missing/censored data handled appropriately?	Y	Y	PY	NI
**D4**	4.4 If class-imbalance correction was used, predictions recalibrated appropriately?	NA	NA	NA	NA
**D4**	4.5 Methods used to limit potential overfitting?	PY	Y	PY	Y

Domain and overall judgments - model development

Report	D1	D2	D3	D4	Overall development quality	Applicability to review question
**Ma et al. [11]**	Low	Low	Low	High	High concern	Low concern
**Lin et al. [12]**	Low	Unclear	Low	High	High concern	High concern
**Yuan et al. [13]**	Low	Unclear	Low	High	High concern	Low concern
**Ding et al. [14]**	Low	Low	Low	High	High concern	Low concern

Panel C. PROBAST+AI model-evaluation risk-of-bias assessment [22].

Domain	Signaling question	Ma et al. [11]	Lin et al. [12]	Yuan et al. [13]	Ding et al. [14]
**D1**	1.1 Appropriate data sources?	Y	Y	Y	Y
**D1**	1.2 Appropriate study design?	Y	Y	Y	Y
**D1**	1.3 Inclusion/exclusion yielded a representative evaluation dataset?	Y	PY	PY	PY
**D2**	2.1 Predictors defined and assessed similarly for all participants?	Y	Y	Y	Y
**D2**	2.2 Predictor preprocessing applied consistently?	Y	Y	Y	Y
**D2**	2.3 Predictor assessment performed without knowledge of the outcome?	Y	NI	NI	Y
**D2**	2.4 Predictors available at the intended time of model use?	Y	Y	Y	Y
**D3**	3.1 Outcome defined and assessed appropriately?	Y	Y	Y	Y
**D3**	3.2 Outcome defined and assessed similarly for all participants?	Y	Y	Y	Y
**D3**	3.3 Outcome assessed without knowledge/use of predictors?	PY	Y	PY	Y
**D3**	3.4 Predictor-outcome interval appropriate?	Y	Y	PY	Y
**D4**	4.1 Evaluation not based only on apparent performance?	Y	PN	Y	Y
**D4**	4.2 Evaluation sample size reasonable?	N	PY	PN	PN
**D4**	4.3 Missing/censored data handled appropriately?	Y	Y	PN	NI
**D4**	4.4 If class-imbalance correction was used, evaluation conducted appropriately?	NA	NA	NA	NA
**D4**	4.5 If data splitting was used, evidence leakage avoided?	NA	PN	NA	PN
**D4**	4.6 If resampling was used, all model-development steps repeated within resampling?	PN	NA	N	NA
**D4**	4.7 Predictive performance evaluated appropriately?	PN	PN	PY	Y

Domain and overall judgments - model evaluation

Report	D1	D2	D3	D4	Overall evaluation risk of bias	Applicability to review question
**Ma et al. [11]**	Low	Low	Low	High	High risk	Low concern
**Lin et al. [12]**	Low	Unclear	Low	High	High risk	Low concern
**Yuan et al. [13]**	Low	Unclear	Low	High	High risk	Low concern
**Ding et al. [14]**	Low	Low	Low	High	High risk	Low concern

Assessment notes: For Lin et al. [12], model development and formal external evaluation were performed in the complete mixed HNSCC cohort; the review-relevant oral SCC AUC was reported as a subgroup estimate and was not separately reported for an independent oral-only external validation set. For Ma et al. [11], the report did not clearly establish that all feature-selection steps were repeated within each LOOCV iteration. For Yuan et al. [13], the published workflow did not establish that clustering, preprocessing, imputation, and feature selection were fully nested within each cross-validation fold. For Ding et al. [14], the random 7:3 split was repeated until baseline characteristics were approximately balanced, so the held-out test partition was not selected entirely independently of inspected cohort characteristics.

Abbreviations and symbols: AI, artificial intelligence; D1, participants and data sources; D2, predictors; D3, outcome; D4, analysis; HNSCC, head and neck squamous cell carcinoma; LOOCV, leave-one-out cross-validation; MPR, major pathological response; NA, not applicable; NI, no information; OSCC, oral squamous cell carcinoma; PN, probably no; PROBAST+AI, Prediction model Risk Of Bias ASsessment Tool plus artificial intelligence; PY, probably yes; QUIPS, Quality In Prognosis Studies; ROC, receiver operating characteristic; SCC, squamous cell carcinoma; Y, yes; N, no. QUIPS domain ratings: Low, Moderate, or High risk of bias. PROBAST+AI domain/overall ratings: Low, High, or Unclear concern for model-development quality and Low, High, or Unclear risk of bias for model evaluation. Applicability is judged as Low, High, or Unclear concern. A negative signaling-question response does not automatically determine the domain judgment; judgments integrate all signaling questions and study context.
