# Supplementary material for: Computational and AI-Enabled Imaging Biomarkers for Predicting and Assessing Immunotherapy Response in Oral Squamous Cell Carcinoma: A Systematic Review with Functional Meta-Synthesis
Source: Med Sci (Basel). 2026 Sep 8;14(5):554. doi: 10.3390/medsci14050554 (PMC13609177; doi:10.3390/medsci14050554)
Supplement: Supplementary file 1 [file medsci-14-00554-s001.zip › Supplementary Table S6 METRICS Assessment.pdf]

Supplementary Table S6. METRICS assessment of radiomics and machine-learning studies

Item-level methodological-quality assessment using METRICS [24]. The final percentage score was normalized to the maximum achievable score after accounting for conditional items.

No.	METRICS item	Weight	Ma et al. [11]	Lin et al. [12]	Yuan et al. [13]	Ding et al. [14]
1	Adherence to a radiomics/ML-specific guideline or checklist	0.0368	No	No	Yes	No
2	Eligibility criteria describe a representative study population	0.0735	Yes	Yes	Yes	Yes
3	High-quality reference standard with a clear definition	0.0919	Yes	Yes	Yes	Yes
4	Multicenter design/data	0.0438	No	Yes	Yes	No
5	Clinically translatable imaging source	0.0292	Yes	Yes	Yes	Yes
6	Imaging acquisition protocol reported sufficiently	0.0438	No	Yes	Yes	Yes
7	Appropriate interval between imaging and reference standard	0.0292	Yes	Yes	No	Yes
8	Transparent segmentation methodology	0.0337	Yes	Yes	Yes	Yes
9	Formal evaluation of fully automated segmentation (conditional)	0.0225	N/A	N/A	N/A	N/A
10	Test-set segmentation masks produced by a single reader or automated tool	0.0112	No	No	No	No
11	Appropriate image preprocessing with transparent parameters	0.0622	No	Yes	No	No
12	Standardized feature-extraction software/version (handcrafted radiomics)	0.0311	Yes	Yes	No	Yes
13	Transparent feature-extraction parameters/configuration	0.0415	No	Yes	No	No
14	Removal of non-robust features (tabular radiomics)	0.0200	No	Yes	No	Yes
15	Removal of redundant features	0.0200	Yes	Yes	Yes	Yes
16	Dimensionality appropriate relative to data size	0.0300	No	Yes	No	Yes
17	Robustness assessment of end-to-end deep-learning pipeline (conditional)	0.0200	N/A	N/A	N/A	N/A
18	Proper early data partitioning and leakage prevention	0.0599	No	Yes	No	No
19	Potential confounding handled appropriately	0.0300	No	Yes	No	Yes
20	Appropriate task-performance metrics	0.0352	No	No	Yes	No
21	Uncertainty of performance estimates considered	0.0234	No	Yes	Yes	Yes
22	Calibration assessed	0.0176	No	Yes	Yes	Yes
23	Uniparametric imaging adequate or added value of multiparametric imaging shown	0.0117	Yes	No	Yes	Yes
24	Non-radiomic comparator or added clinical value assessed	0.0293	Yes	Yes	No	Yes
25	Comparison with simple/classical statistical models	0.0176	Yes	Yes	Yes	Yes
26	Independent internal test using data from the same source	0.0375	No	Yes	No	Yes
27	External test using data from another institution/source	0.0749	No	Yes	No	No
28	Public data availability	0.0075	No	Yes	No	No
29	Public code availability	0.0075	No	No	No	No
30	Public model availability	0.0075	No	No	No	No

Weighted total scores

Report	METRICS score (%)	Quality category
**Ma et al. [11]**	38.3	Low
**Lin et al. [12]**	88.5	Excellent
**Yuan et al. [13]**	49.9	Moderate
**Ding et al. [14]**	59.5	Moderate

Key assessment notes: Ma et al. [11] used LOOCV after feature reduction, but the report did not establish a fully nested feature-selection pipeline, did not report calibration or uncertainty intervals for model performance, and did not use an independent test or external cohort. Lin et al. [12] used multicenter training, internal-validation, and external-validation cohorts, reported feature-reproducibility filtering, calibration, DCA, and publicly available data; however, the external validation concerned the overall HNSCC model rather than an independently validated oral-only model. Yuan et al. [13] followed TITAN/REMARK, used multicenter MRI and five-fold cross-validation, and reported uncertainty and calibration; the report did not establish that clustering, imputation, and feature selection were fully nested within each fold and did not use an independent center-separated external test. Ding et al. [14] used an internal held-out test set, calibration, DCA, and feature-stability filtering; however, the 7:3 split was repeated until baseline characteristics were approximately balanced and no external test cohort was used.

Scoring notes: Items 9 and 17 were not applicable to these four handcrafted/tabular radiomics pipelines and were removed from the achievable-score denominator. “No” indicates that the METRICS criterion was not demonstrably fulfilled from the published report and available supplementary material; it does not necessarily indicate that the procedure was not performed. The METRICS authors categorize scores as very low (0 to <20%), low (20 to <40%), moderate (40 to <60%), good (60 to <80%), and excellent (80 to 100%) and recommend that the category complement, rather than replace, the quantitative score.

Abbreviations: AI, artificial intelligence; DCA, decision curve analysis; DL, deep learning; HNSCC, head and neck squamous cell carcinoma; LOOCV, leave-one-out cross-validation; METRICS, METhodological RadiomICs Score; ML, machine learning; MRI, magnetic resonance imaging; N/A, not applicable; OSCC, oral squamous cell carcinoma; REMARK, Reporting Recommendations for Tumor Marker Prognostic Studies; TITAN, Transparency in the Reporting of Artificial Intelligence; Yes, criterion fulfilled; No, criterion not demonstrably fulfilled.
