# Supplementary material for: Computational and AI-Enabled Imaging Biomarkers for Predicting and Assessing Immunotherapy Response in Oral Squamous Cell Carcinoma: A Systematic Review with Functional Meta-Synthesis
Source: Med Sci (Basel). 2026 Sep 8;14(5):554. doi: 10.3390/medsci14050554 (PMC13609177; doi:10.3390/medsci14050554)
Supplement: Supplementary file 1 [file medsci-14-00554-s001.zip › Supplementary Table S7 .pdf]

Supplementary Table S7. Prospective registration, protocol availability, and reporting-bias assessment of the included reports.

| Study | Trial registration/protocol | Timing/status of registration | Prespecification relevant to imaging-response analysis | Discrepancies identified | Reporting-bias assessment |
|---|---|---|---|---|---|
| Shah et al. [8] | NCT02919683 | Parent trial prospectively registered; imaging report was a secondary retrospective analysis | Parent trial documented; detailed FDG-PET/pathological-response analysis plan not identified | No overt outcome discrepancy identified | Selective analytical reporting could not be excluded |
| Wondergem et al. [9] | EudraCT 2018-002643-28; NeoNivo protocol | Prospectively registered | PET measurements, longitudinal uptake changes, response and biomarker relationships prespecified | No overt discrepancy identified | Lower concern based on available prospective documentation |
| Jiang et al. [10] | NCT05034146; NCT05030597 | Registered clinical protocols | FAPI-PET response analysis described as a prespecified exploratory aim | No overt response-outcome discrepancy identified | Selective analytical reporting could not be fully excluded |
| Ma et al. [11] | None identified for the radiomics analysis | Not applicable; no prospective study-specific registration or radiomics-analysis protocol identified | No prospective radiomics-analysis plan identified | Could not be assessed against a prospective plan | Selective analytical reporting could not be reliably excluded |
| Lin et al. [12] | No study-specific prospective radiomics protocol identified | Not applicable; no prospective study-specific registration or radiomics-analysis protocol identified | No prospective radiomics-analysis plan identified | Could not be assessed against a prospective plan | Selective analytical reporting could not be reliably excluded |

| Study | Trial registration/protocol | Timing/status of registration | Prespecification relevant to imaging-response analysis | Discrepancies identified | Reporting-bias assessment |
|---|---|---|---|---|---|
| Yuan et al. [13] | NCT06854107 | Registered after completion of the reported retrospective data-collection period | Does not constitute prospective specification of the reported radiomics analysis | Could not be fully assessed prospectively | Selective analytical reporting could not be reliably excluded |
| Ding et al. [14] | NCT05941338; NCT05980702; NCT06130332; NCT04718415 | Registered parent neoadjuvant trials | Treatment/response outcomes traceable; DeltaHabitat analysis not specifically prespecified in an identified prospective analysis plan | No overt clinical-response discrepancy identified | Selective model/analytical reporting could not be excluded |
